# Economic impact of screening for X-linked Adrenoleukodystrophy within a newborn blood spot screening programme

**DOI:** 10.1186/s13023-018-0921-4

**Published:** 2018-10-11

**Authors:** Alice Bessey, James B Chilcott, Joanna Leaviss, Anthea Sutton

**Affiliations:** School of Health and Related Research, The University of Sheffield, Regent Court, 30 Regent Street, Sheffield, S1 4DA UK

**Keywords:** Adrenoleukodystrophy, Neonatal screening, Newborn screening, Cost-benefit analysis, Cost-effectiveness analysis, Economic analysis, Hematopoietic stem cell transplantation, Decision trees

## Abstract

**Background:**

A decision tree model was built to estimate the economic impact of introducing screening for X-linked adrenoleukodystrophy (X-ALD) into an existing tandem mass spectrometry based newborn screening programme. The model was based upon the UK National Health Service (NHS) Newborn Blood Spot Screening Programme and a public service perspective was used with a lifetime horizon. The model structure and parameterisation were based upon literature reviews and expert clinical judgment. Outcomes included health, social care and education costs and quality adjusted life years (QALYs). The model assessed screening of boys only and evaluated the impact of improved outcomes from hematopoietic stem cell transplantation in patients with cerebral childhood X-ALD (CCALD). Threshold analyses were used to examine the potential impact of utility decrements for non-CCALD patients identified by screening.

**Results:**

It is estimated that screening 780,000 newborns annually will identify 18 (95%CI 12, 27) boys with X-ALD, of whom 10 (95% CI 6, 15) will develop CCALD. It is estimated that screening may detect 7 (95% CI 3, 12) children with other peroxisomal disorders who may also have arisen symptomatically. If results for girls are returned an additional 17 (95% CI 12, 25) cases of X-ALD will be identified. The programme is estimated to cost an additional £402,000 (95% CI £399–407,000) with savings in lifetime health, social care and education costs leading to an overall discounted cost saving of £3.04 (95% CI £5.69, £1.19) million per year. Patients with CCALD are estimated to gain 8.5 discounted QALYs each giving an overall programme benefit of 82 (95% CI 43, 139) QALYs.

**Conclusion:**

Including screening of boys for X-ALD into an existing tandem mass spectrometry based newborn screening programme is projected to reduce lifetime costs and improve outcomes for those with CCALD. The potential disbenefit to those identified with non-CCALD conditions would need to be substantial in order to outweigh the benefit to those with CCALD. Further evidence is required on the potential QALY impact of early diagnosis both for non-CCALD X-ALD and other peroxisomal disorders. The favourable economic results are driven by estimated reductions in the social care and education costs.

**Electronic supplementary material:**

The online version of this article (10.1186/s13023-018-0921-4) contains supplementary material, which is available to authorized users.

## Background

X-linked adrenoleukodystrophy (X-ALD) is a rare genetic disorder caused by a defect in the ABCD1 gene. The disorder interrupts peroxisomal fatty acid beta oxidation resulting in the accumulation of very long chain fatty acids with consequent damage to tissue throughout the body and brain. X-ALD demonstrates X-linked recessive inheritance, with reports of incidence varying between 0.8 and 4.76 people affected per 100,000 births [1–3].

Males with X-ALD can present with adrenal insufficiency, the cerebral form of X-ALD, or progressive myelopathy (adrenomyeloneuropathy (AMN)). The majority of men with X-ALD will go on to develop AMN. Most women with X-ALD will also develop symptomatic AMN over their lifetime, but women do not appear to be affected by cerebral deterioration or adrenal insufficiency [4–7]. Cerebral X-ALD is the most severe phenotype and without treatment patients may experience neurodegenerative decline leading to a vegetative state and death. Studies have shown that haematopoietic stem cell transplantation (HSCT) and more recently gene therapy can be successful in preventing long term deterioration in patients with cerebral X-ALD presenting in childhood or adolescence (CCALD) [8–11]. However, this benefit is dependent on being transplanted at the first signs of neurological development with little or no benefit to patients transplanted after this point [9–11]. There is also emerging evidence of similar benefits in early transplanted adults [12].

Patients currently undergoing HSCT before the onset of significant neurological symptoms, referred to as early HSCT are most frequently identified due to family history or through presenting with adrenal insufficiency. While screening of the extended family of X-ALD patients is currently offered it is estimated that between 5 and 18% of new patients present with a spontaneous mutation [13, 14]. A population screening approach using existing newborn blood spots and high throughput tandem mass spectrometry has been shown to accurately diagnose affected individuals and to have a high sensitivity and specificity [13, 15]. The aim of screening is to identify patients before they become symptomatic to enable them to be monitored for the initial signs of CCALD and transplanted at an optimal time. This method of screening has been in use in New York State since 2014 and has been found to identify patients with other peroxisomal disorders as well as X-ALD [16, 17].

Screening programmes use criteria, often based on the Wilson and Jungner criteria, to decide which conditions should be screened for. These criteria often incorporate an economic component, for example, the Wilson and Jungner criteria state that the cost of case-finding, including diagnosis and treatment of all patients diagnosed should be economically balanced in relation to possible expenditure on medical care as a whole [18]. In the United Kingdom (UK), the National Screening Committee (NSC) criteria describe this economic requirement in terms of the potential cost-effectiveness of a screening technology [19]. The aim of this study was to address this criterion and estimate the potential cost-effectiveness of including screening for X-ALD in the UK National Health Service (NHS) Newborn Blood Spot Screening Programme.

## Methods

**Fig. 1:**
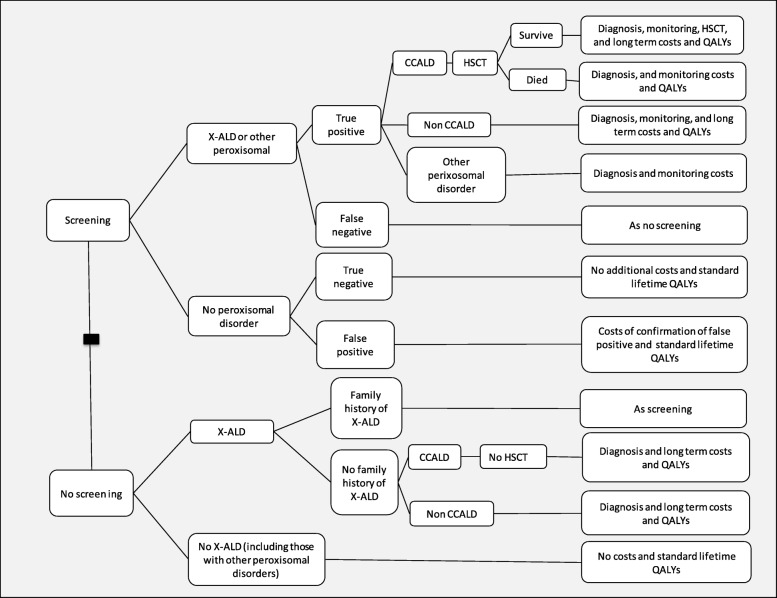
X-ALD Screening Decision Tree

A decision analytic model was built to estimate the economic impact of screening for X-ALD in the NHS Newborn Blood Spot Screening Programme. The model took an NHS and personal social services (PSS) perspective, included a lifetime horizon and discounted costs and benefits at 3.5% [20]. The model estimated life years and quality adjusted life years (QALYs) gained, health, social care and special education costs. Costs were estimated for 2014/15 with uplifting according to hospital and community health services indices [21]. The incremental cost effectiveness ratio (ICER) is defined as the cost per QALY gained. The model used a decision tree structure, shown in Fig. 1 to estimate the number of newborns that might be identified with a positive screening result, the distribution across the peroxisomal disorders and the number developing CCALD and undergoing HSCT with and without screening. It was assumed that the outcomes of early HSCT in patients identified through screening would be equivalent to outcomes in those identified early without screening. Lifetime costs and QALYs for the different outcomes with and without screening were estimated using lifetables. The annual number of births for the UK was estimated based on a 10 year average [22–24]. Patients identified with other peroxisomal disorders were assumed to incur incremental costs of screening and confirmatory diagnosis, but no health benefits or disbenefits were associated with early diagnosis from screening. Details of all model parameters are given in Table 1 and further details on the model distribution used in the probabilistic sensitivity analysis are given in Additional file 1.

**Table 1:** Parameters Table

Parameter	Mean (95% Confidence Interval)	Reference
Base case parameters		
Number of births per year	400,308	[22–24]
X-ALD incidence	1 in 22,361 (15,083, 33,153)	[3, 29–32]
Proportion CCALD, AMN, Addison’s/Asymptomatic	0.53 (0.36, 0.69), 0.32 (0.18, 0.49), 0.15 (0.05, 0.28)	[14]
Non-X-ALD peroxisomal incidence	1 in 63,000 (33,897, 117,090)	[32]
Age at presentation CCALD	7 (6.76, 7.24)	[11]
Survival from onset CCALD Weibull distribution - shape parameter - scale parameter - correlation	ᅟ ᅟ −2.970 0.162 −0.8994	[11]
Time to CCALD progression (years)	1.6 (1.34, 1.86)	[11]
Mortality risk HSCT	0.08 (0.01, 0.21)	[10]
Proportion of CCALD currently undergoing early transplant (Family history)	0.33 (0.23, 0.43)	[10]
Proportion ALD-DRS 0, ALD-DRS1, ALD-DRS2, ALD-DRS 3–4, after HSCT	0.62 (0.35, 0.85), 0.23 (0.05, 0.48), 0.08 (0.002, 0.26), 0.08 (0.002, 0.26)	[10]
Proportion successful HSCT develop AMN	0.6 (0.19, 0.93)	[65]
Sensitivity	0.995	
Specificity	1	
Proportion of AMN mild	0.51 (0.38, 0.64)	[44]
Proportion of AMN developing adult onset cerebral X-ALD	0.63 (0.44, 0.8)	[40]
Age at presentation AMN (years)	35.3 (26.7, 43.9)	[40]
Time to development of adult onset cerebral X-ALD (years)	10.2 (3.3, 17.1)	[40]
Survival adult onset cerebral X-ALD (years)	3.4 (0.5, 6.3)	[40]
QALYs	See Additional file 2	
Costs	See Additional file 3	

A comprehensive, systematic search of bibliographic databases was conducted to identify literature on X-ALD to inform model structure and parameterisation. Information requirements were defined prospectively, however data searching and data extraction remained dynamic in order to reflect additional information needs identified during model development [25]. Full details of evidence searches and reviews are provided in the Additional file 2.

The NHS Newborn Blood Spot Screening Programme already uses tandem mass spectrometry hence the incremental cost of including testing for X-ALD is small and was estimated at £0.50 per baby based on a previous economic evaluation [26]. The review identified four studies that reported the sensitivity and specificity of newborn blood spot tandem mass spectrometry for X-ALD. All studies showed either 100% sensitivity or 100% specificity or both [13, 15, 27, 28]. However, as false negatives are likely in a population based screening programme we assumed a sensitivity of 99.5%.

**Fig. 2:**
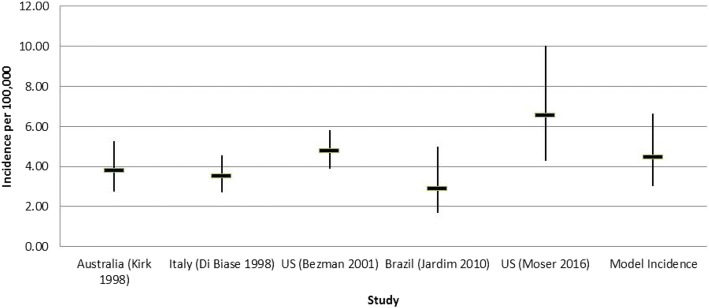
Forest Plot of Incidence Values. The black horizontal line represents the mean incidence and the black vertical line the 95% confidence interval

Incidence of X-ALD from four studies identified in the review and an additional study identified after the review that included data on incidence from the New York screening programme were synthesised using a random effects model in WinBugs [3, 29–32]. Studies were included that gave both the number of cases and relevant population figure, retrospective studies that included both sexes fatty acid measurements were excluded due to historic underreporting of X-ALD in women and studies that included cases from before the adoption of very long-chain fatty acid measurements were also excluded as they are likely to only report a minimum estimates [33]. The Moser et al. study [32] was included as it provided an important estimate of the incidence of X-ALD once a screening programme has been implemented of both X-ALD and the other peroxisomal conditions that are identified. The incidence values from the five studies [3, 29–32] and the synthesised incidence of 1 in 22,000 (95%Confidence Interval (CI) 1 in 33,000, 1 in 15,000) used in the model are shown in a forest plot in Fig. 2. There is no direct evidence concerning the incidence of the other peroxisomal conditions that might be identified by screening in the UK, we therefore used the incidence of 1 in 63,000 (95% CI 1 in 117,000, 1 in 34,000) from the New York Screening Programme [32].

The review identified 10 studies that estimated the phenotype breakdown of X-ALD [2, 14, 29, 31, 34–39]. The studies were heterogeneous and differed in terms of how the phenotypes were classified. Due to this no attempt was made to synthesise evidence, rather the Horn et al. [14] study of X-ALD in Norway was chosen on the basis of study quality and relative generalisability to the UK population. This assumption was tested in a sensitivity analysis. The study of AMN by de Beer et al. [40] was used to estimate the proportion of AMN patients that go on to develop cerebral involvement.

The review identified 26 studies on either the natural history or outcome following transplantation [6, 9–11, 35, 40–62]. For patients with CCALD, there is no direct comparative evidence on survival with and without HSCT. Survival and time to progression without transplantation were estimated from the Mahmood et al. study [11], selected from the 26 studies [6, 9–11, 35, 40–62] as it presented data on a large cohort, 283 patients, with follow-up of up to 30 years. The model assumed that CCALD patients start off with mild to moderate disease before progressing to severe CCALD indicated by developing two or more neurological deficits with a mean time to progression of 1.6 years. The mean survival in these patients of 19 years (16.8–21) was estimated using simulated patient level data from the Mahmood study [11, 63] with parametric extrapolation of long term survival according to the methods recommended by the National Institute for Health and Care Excellence (NICE) Decision Support Unit [64].

For those undergoing HSCT the Peters et al. [10] study was selected from the 26 studies identified [6, 9–11, 35, 40–62] as it provided detailed outcomes following HSCT, including the ALD-Disability rating scale (ALD-DRS) and provided survival and outcomes data based on the severity of the disease at transplant. The model assumed that only patients with a Loes score of < 10 undergo HSCT and that those who survive and have a good outcome following HSCT have a normal life expectancy as no deaths occurred post 1.5 years for those undergoing early HSCT [9, 10]. The proportion of patients identified through a family history without screening in the Peters et al. [10] study was used to estimate the number of those diagnosed early enough to undergo HSCT. In the screen arm it was assumed that all CCALD patients undergo HSCT. This assumption was explored in a sensitivity analysis.

Estimates of patient outcomes following HSCT were based on their functional level assessed by the ALD-DRS before and after HSCT [10]. The outcomes of patients with an ALD-DRS rating of 0 before HSCT were used to model outcomes following successful HSCT [10]. Yearly costs and QALYs were applied to the different disability levels to estimate long term outcomes. A normal life expectancy was assumed for those with an outcome of ALD-DRS level 0–2 and life expectancy of non-transplanted CCALD for those with an outcome of ALD-DRS of 3–4.

HSCT does not prevent myelopathy in adulthood. In a small study of five patients who had undergone successful HSCT for CCALD [65], age range 18–25, three patients developed myelopathy. This rate of development of AMN type symptoms was applied to those who had undergone transplantation and whose outcomes were either ALD-DRS 0 or 1. A lower age of symptom onset (20) was used for these patients in line with the study. Additional costs and QALY decrements were incurred in line with AMN patients.

The model assumed normal survival for AMN and progression per se is not modelled but patients are split between a mild or moderate/severe form from the onset of the disease. The model parameters were based on a study with a cohort of 60 men that provided expanded disability status scale (EDSS) used to estimate quality of life and costs [44]. A second study by de Beer et al. [40] was used to estimate a number of parameters for AMN and adult onset cerebral X-ALD that were not provided in the Keller et al. study [44] (see Table 1). The model assumed that the same proportion of both the mild and moderate/severe AMN patients go on to develop adult onset cerebral X-ALD.

Asymptomatic and Addison’s only cases were assumed to have normal life expectancy and morbidity. It is also assumed that the Addison’s/Asymptomatic cases are monitored from birth in both arms but that all AMN cases are diagnosed symptomatically in the no-screen arm.

No studies were identified providing direct evidence on quality of life utilities for X-ALD patients; furthermore no suitable proxy condition was identified for CCALD. Age specific general UK population QALYs were used for pre-symptomatic patients, those with Addison’s only, and for those with ALD-DRS of 0 following transplant [66]. Based on the description of the ALD-DRS each state was assigned an equivalent EuroQol Five Dimension Five Level (EQ-5D-5 L) health state, shown in Additional file 3 [67]. QALYs for mild/moderate CCALD were calculated as the average of ALD-DRS1 and 2 and the QALYs for moderate/severe CCALD were calculated as the average of ALD-DRS 3 and 4.

For patients with AMN and women with X-ALD multiple sclerosis (MS) was used as a proxy as the Expanded Disability Status Scale (EDSS) has been used in both patient populations [40, 44]. The difference between the mean value for the EDSS state 3 (mild AMN) and EDSS state 6.5 (moderate/severe AMN) and the general population norms were calculated and proportionate differences were then applied to the age specific general population norms to give the utility decrements for each age group [66, 68]. Further detail on the calculations of the QALYs is provided in Additional file 3.

The costs of monitoring, diagnosis and a yearly cost of management were estimated for each phenotype. This was an iterative process that involved developing a resource use profile based on published guidelines and guidance for patients and families with X-ALD [5, 16]. The resource use profiles were sent out for consultation by ALD Life. The feedback from this process was used to create final resource use descriptions presented in Additional file 4 and were costed using appropriate sources [21, 69–71].

The cost of diagnosis in symptomatic patients included GP and specialist appointments associated with the increased diagnostic journey over and above the standard tests and consultations for all diagnosed patients as outlined in the Additional file 4. Untreated CCALD results in substantial disability with consequent high social care costs and education costs. In the experiences of the users of ALD Life all CCALD patients require education support often at the 1:1 or 2:1 level. Special education costs have been estimated based on the costs of education of children with autism who attended, in line with patients with CCALD, mainstream, special, and residential schools. The uplifted yearly cost is around £24,000 [72]. Social and education costs are not separated in the model as care packages can include funding for special education and social care. Social care costs included respite care and equipment but not the costs of home adaptation. For those over 18 years just the social care package and no education costs were included. Non-medical costs such as aids and home help and transportations for AMN and women with X-ALD were taken from a study on MS which provided costs for EDSS state 2 and EDSS 6.5 which were used for mild AMN and moderate/severe AMN respectively. The costs for EDSS state 2 were also used for the women with X-ALD in the corresponding age group of 40+ [6, 44, 68, 73]. Details of the costs are provided in Additional file 4.

Input parameters were characterised probabilistically, see Additional file 1, and uncertainty was propagated with Monte Carlo sampling with 100,000 replicates in the base case and 10,000 replicates in the sensitivity analyses. A number of one-way sensitivity analyses were undertaken to explore the impact of assumptions and structural uncertainties in the model.

1. The proportion of patients who develop CCALD may be overestimated as they are the cases most classically associated with X-ALD and there were differences between the identified studies [2, 14, 29, 31, 34–39]. The proportion of X-ALD patients that develop CCALD was decreased to 10%, 15%, and 20%. In each case it was estimated, based on the base case inputs, that 69% of non-CCALD X-ALD was AMN, and 31% was Addison’s only/Asymptomatic.
2. The proportion of CCALD patients in the screen arm who undergo HSCT was reduced from 100 to 60%
3. Scenario analyses were conducted that varied both the proportion of patients who developed CCALD and the proportion of CCALD patients in the screen arm who undergo HSCT
4. Both sexes were screened for. It is assumed based on data from the New York Screening Programme that the incidence in females is the same as that in males [7, 32]. For this sensitivity analysis the study by Engelen et al. [6] was used to model progression of the disease in women. It was assumed that the disease progressed with age and that all women would become affected. EDSS scores were converted into EQ-5D quality of life scores and the average score for three age groups 18–39, 40–59, and 60+ years were used in the model [6, 68, 73].
5. NICE specifies that a lower discount rate of 1.5% can be used for public health interventions [20]. A sensitivity analysis using the lower discount rate was also conducted.
6. As there is some uncertainty as to the incidence rate in the UK the incidence rate was doubled and halved in order to explore the impact on the results.
7. Patients or parents of patients with non-CCALD X-ALD and those with other non-X-ALD disorders may experience a disbenefit from a positive screen results. For those with non-CCALD they or their parents may experience anxiety about the potential for developing CCLAD or developing the non-treatable AMN. Patients and parents of those with who test positive for other non X-ALD peroxisomal disorders may also experience anxiety or distress from being diagnosed before they become symptomatic. It is unclear how these disbenefits would present and if they would be limited to anxiety or if they would present in other behavioural changes. In order to explore and try to quantify this uncertainty an exploratory threshold analysis was undertaken that explored the maximum disbenefit, expressed in QALYs, per patient per year that non-CCALD identified patients would need to experience in order to cancel out the benefits that accrue to CCALD patients due to screening. The number of cases is multiplied by the length of time they would be expected to be asymptomatic. For non CCALD X-ALD this is assumed to be the age of onset of AMN (35 years), for the non-X-ALD cases we have assumed that is will be 5 years, and for woman with X-ALD we have assumed that is will be 50 years.

## Results

The results of including screening for X-ALD in the NHS Newborn Blood Spot Screening Programme are presented in Tables 2 and 3. It is estimated that screening an annual UK birth cohort of approximately 780,000 newborns would identify 18 (95%CI 12, 27) males with X-ALD. It is expected that 6 (95% CI 3, 10) of these newborns will develop AMN, with 10 (95% CI 6, 15) progressing to CCALD, and approximately 3 (95% CI 0.9, 6) having Addison’s only or being asymptomatic. The model also estimates that screening will detect 7 (95% CI 3, 12) cases of other peroxisomal disorders each year. If girls are also screened it will result in an additional 17 (95% CI 12, 25) cases of X-ALD and around 13 (95%CI 7, 23) cases of other peroxisomal disorders in total.

**Table 2:** Model estimated number of X-ALD and CCALD cases per year

Sensitivity Analyses	Number of cases			
	X-ALD Cases	95% Confidence Interval	CCALD Cases	95% Confidence Interval
Base case	18.3	(12.1,26.6)	9.7	(5.5,15.3)
Incidence rate doubled	36.5	(24.,53.2)	19.3	(11.1,31.0)
Incidence rate halved	9.1	(6.,13.2)	4.8	(2.7,7.6)
CCALD 20% of total X-ALD	–	–	3.6	(2.4,5.3)
CCALD 15% of total X-ALD	–	–	2.7	(1.8,4.)
CCALD 10% of total X-ALD	–	–	1.8	(1.2,2.7)
Both girls and boys screened	35.6	(23.7,51.9)	–	–

– Number of cases are the same as the base case

**Table 3:** Cost-effectiveness results

Sensitivity analyses	Screening		No screening		Incremental				
	Total Costs (*m*)	Total QALYs	Total Costs (*m*)	Total QALYs	Costs (*m*)	95% Confidence Interval (*m*)	QALYs	95% Confidence Interval	ICER
Base case	£3.01	390	£6.44	307	-£3.04	(−£5.69, −£1.19)	82	(43, 139)	Dominates
Incidence rate doubled	£5.97	778	£12.88	614	-£6.50	(−£11.74, −£2.80)	164	(86, 277)	Dominates
Incidence rate halved	£1.51	194	£3.21	153	-£1.30	(−£2.62, −£0.39)	41	(22, 69)	Dominates
CCALD 10% of total X-ALD	£2.02	412	£2.21	397	£0.21	(−£0.06, £0.46)	16	(9, 24)	£13,600
CCALD 15% of total X-ALD	£2.13	409	£2.70	386	-£0.17	(−£0.63, £0.22)	23	(14, 36)	Dominates
CCALD 60% HSCT rate	£5.20	340	£6.41	307	-£0.81	(−£2.01, £0.02)	33	(15, 60)	Dominates
CCALD 10% of total X-ALD and 80% HSCT rate	£2.23	407	£2.21	396	£0.42	(£0.24, £0.62)	11	(6, 17)	£38,701
CCALD 15% of total X-ALD and 80% HSCT rate	£2.43	401	£2.69	385	£0.15	(−£0.18, £0.42)	16	(9, 26)	£8927
CCALD 20% of total X-ALD and 80% HSCT rate	£2.65	396	£3.18	375	-£0.12	(−£0.59, £0.24)	22	(13, 34)	Dominates
1.5% Discount Rate	£4.59	611	£9.35	455	-£4.36	(−£7.97, −£1.81)	156	(83, 260)	Dominates
Both girls and boys screened	£3.27	800	£6.96	718	-£3.27	(−£5.97, −£1.36)	82	(43, 139)	Dominates

*m* million

Adding X-ALD to the screening programme as a whole results in an increase in total discounted QALYs and life years per year of 82 (95% CI 43, 139) and 79 (95% CI 42, 131) respectively. The increase in QALYs and life years is due to improvements in the outcomes of patients with CCALD only who on average have a gain of 8.5 QALYS per CCALD patient and a life year gain of just over 8 years per CCALD patient.

The screening programme is estimated to cost an additional £402,000 (95% CI £399–407,000) per year with discounted marginal lifetime health and social care / education costs of £256,000 (95% CI £12,000, £527,000) and -£3.69 (95% CI -£6.27, −£1.92) million respectively, leading to an overall discounted cost saving of £3.04 (95% CI £5.69, £1.19) million per year of screening.

**Fig. 3:**
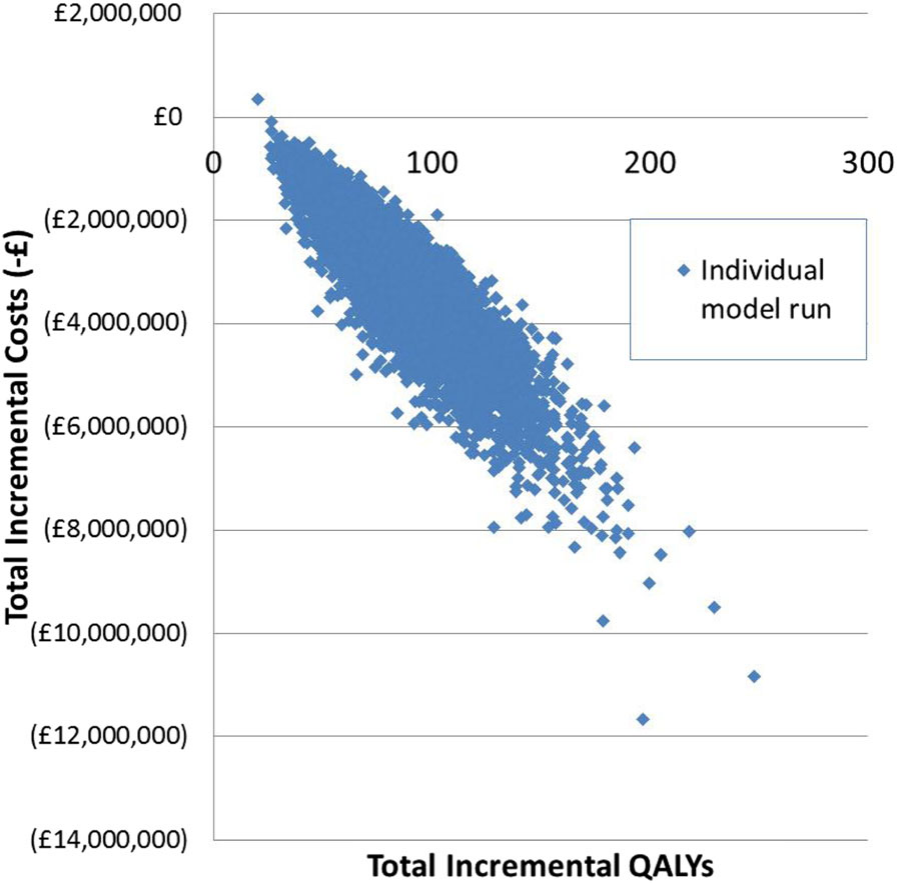
Cost-Effectiveness Plane. Each of the blue diamonds represents one of the 100,000 model runs. Costs in () represent model runs where screening is estimated to be cost saving

As screening is estimated to result in more QALYs and fewer costs, screening is said to dominate no screening. The results of the probabilistic sensitivity analysis are shown in the cost-effectiveness plane in Fig. 3. In the base case screening dominates no screening. This means that adding X-ALD screening into the existing screening programme results in additional QALYs gained and lower total discounted costs.. Across the sensitivity analyses screening dominated no screening except when the proportion of X-ALD that developed CCALD was reduced to 10%. The ICER went above the threshold of £30,000 per QALY when both the proportion of X-ALD developing CCALD was reduced to 10% and the proportion of CCALD patients receiving an HSCT was reduced to 80%.

The results of the disbenefit analysis are shown in Table 4. Results are shown for both undiscounted QALYs and a discounted analysis which used the total discounted incremental QALYs and discounted age at symptomatic presentation. In the male babies only screened analysis the disbenefit to the non CCALD patients would have to be a relatively substantial 0.84 or 0.40 per case per year in the undiscounted or discounted analysis respectively in order to wipe out the benefit CCALD patients derive from screening. If both sexes were screened the disbenefit to the non CCALD patients that would wipe out the benefit to the CCALD patients would need to be 0.13 in the discounted analysis.

**Table 4:** Results of the Disbenefit Analysis

	Male babies only screened		Both sexes screened	
	Undiscounted	Discounted	Undiscounted	Discounted
Non CCALD X-ALD	8.61	8.61	8.61	8.61
Age of symptomatic presentation	35.00	20.55	35.00	20.55
Person years without diagnosis	301.34	176.95	301.34	176.95
Non X-ALD disorders	6.68	6.68	13.04	13.04
Age of symptomatic presentation	5.00	4.66	5.00	4.66
Person years without diagnosis	33.39	31.11	65.21	60.76
Number of cases of X-ALD in females	–	–	17.36	17.36
Age of symptomatic presentation	–	–	50.00	24.11
Person years without diagnosis	–	–	867.96	418.60
Total person years without diagnosis	334.73	208.06	1234.52	656.32
Total incremental QALYs from screening	280.15	82.42	279.78	82.30
Maximum QALY decrement per non CCALD case per year	0.84	0.40	0.23	0.13

– No cases in females when only male babies are screened

## Discussion

This study has attempted to address whether screening for X-ALD is cost-effective and so fulfils the economic criterion for a screening programme. The study suggests that screening for X-ALD is cost saving, however problems with measurement and valuation of some of the key benefits and harms of screening mean that it is difficult to capture the full scope of these within an economic model. These benefits and harms also relate to other screening criteria, in particular the evidence of treatment benefit and the evidence on benefits and harms from over diagnosis, overtreatment and uncertain findings [19].

Evidence for treatment benefit for CCALD in screening comes from small observational studies in a non-screening setting. Long term evidence on morbidity is difficult to interpret because of the range of different outcome measures that have been used, together with little evidence on quality of life utilities in both transplanted and non-transplanted patients [9–11]. The approach taken here of mapping the ALD-DRS onto the EQ-5D-5 L is an imperfect solution to the quality of life issue but due to methodological and practical issues with valuing health states in children and in those with cognitive disabilities no suitable proxy conditions were found [74–76]. The QALY estimates produced are in line with recent studies have shown that neurological quality of life outcomes are similar in early transplanted CCALD patients to the general population [77, 78]. There is also a lack of evidence concerning the impact of early X-ALD diagnosis on patients who might go on to develop Addison’s disease.

The evidence demonstrates that transplanting CCALD patients at the first signs of cerebral involvement offers the best outcomes in terms of both survival and morbidity [9–11]. However, there currently exists no way of identifying which X-ALD patients will go on to develop cerebral involvement in childhood [4]. The need to intervene before there are significant symptoms and the lack of other treatment options brings with it the potential for over or under-treatment if there is variation in the implementation of clinical guidelines [79]. This could also be exacerbated if CCALD patients or families do not follow monitoring protocols or do not consent to transplantation, a procedure with potentially severe complications.

The quality of life and behavioural impacts of receiving an early diagnosis of X-ALD particularly for those who do not develop CCALD are also not well understood or valued. This is also the case for those diagnosed with other peroxisomal disorders. This is particularly relevant as despite recent studies investigating transplantation in adult onset cerebral ALD, there are no established treatment options for non-CCALD patients and therefore screening is currently unlikely to improve clinical outcomes in these patients outside of improved adrenal monitoring [12, 16, 79]. The issue is also particularly relevant if both sexes are screened as this increases the number of non-CCALD patients identified.

Where possible the impact of these issues has been explored through sensitivity, probabilistic sensitivity and threshold analyses. The results of which suggest that the results are robust to the assumptions made in the model. In addition some of these evidence gaps, such as the types and number of other peroxisomal disorders identified, will be addressed by the results coming out of the New York X-ALD screening programme and other implementing sites [16, 79]. In the longer term the existing X-ALD screening programmes will also be able to address other evidence gaps such as the feasibility and efficacy of monitoring, transplantation protocols and disease natural history. Specific studies may also need to be undertaken in order to fully understand the impact of identifying those with non-CCALD, improve the evidence on the quality of life and resource use of those currently living with CCALD and understand the quality of life and resource impact of the diagnostic journey in symptomatic patients.

There are methodological issues associated with measuring and valuing many of the potential benefits and harms of newborn screening. For instance, impacts on families and carers, including future family planning, measuring and valuing quality of life in children, especially those with cognitive disabilities, and estimating the impact on families of incidental findings arising through screening [74–76, 80]. Not all of these issues are covered by current guidelines on economic evaluation of newborn screening interventions [81] and further methodological work is required to improve the quality and scope of future economic evaluations.

## Conclusion

This study estimates that including screening for X-ALD in a tandem MS based screening programme such as the UK NHS Newborn Blood Spot Screening Programme would result in an increase in QALYs and a decrease in total discounted health, social care and education costs. The results are driven by the reduction in social care costs and the increase in QALYs for CCALD patients. Sensitivity analyses suggest that the results are sensitive to the proportion of patients with X-ALD that go on to develop CCALD. Threshold analyses suggest that any potential disbenefits arising for those with non-CCALD conditions would need to be substantial in order to outweigh the benefit to those with CCALD. However the uncertainties associated with measuring and valuing the benefits and harms of screening in the X-ALD population need to be addressed in order to fully demonstrate that the economic criteria can be fulfilled.

## Additional files

Additional file 1: Full parameters table. This file contains the full parameters table including the distributions used in the probabilistic sensitivity analysis. (DOCX 30 kb)

Additional file 2: Systematic review of ALD for model parameters. This file contains the full methods and results of the systematic review that was carried out to inform the model parameters presented in this manuscript. (DOCX 315 kb)

Additional file 3: Calculation of the Quality Adjusted Life Years (QALYs). This file presents more detail on the calculations that were used to calculate the QALYs in the model presented in this manuscript. (DOCX 27 kb)

Additional file 4: Calculation of the costs and their sources. This file contains a table that outlines the included costs for the model presented in this manuscript and their sources. (DOCX 48 kb)

## Abbreviations

ALD-DRS: Adrenoleukodystrophy Disability Rating Scale
AMN: Adrenomyeloneuropathy
CCALD: Childhood cerebral adrenoleukodystrophy
CI: Confidence interval
EDSS: Expanded Disability Status Scale
EQ-5D-5 L: EuroQol Five Dimension Five Level
HSCT: Haematopoietic stem cell transplantation
ICER: Incremental cost effectiveness ratio
MS: Multiple sclerosis
NHS: National Health Service
NICE: National Institute for Health and Care Excellence
NSC: National Screening Committee
PSS: Personal social services
QALY: Quality adjusted life years
UK: United Kingdom
X-ALD: X-linked adrenoleukodystrophy
