# Supplementary material for: Economic impact of screening for X-linked Adrenoleukodystrophy within a newborn blood spot screening programme
Source: Orphanet J Rare Dis. 2018 Oct 11;13:179. doi: 10.1186/s13023-018-0921-4 (PMC6182830; doi:10.1186/s13023-018-0921-4)
Supplement: Supplementary file 1 — Full parameters table. This file contains the full parameters table including the distributions used in the probabilistic sensitivity analysis. (DOCX 30 kb) [file 13023_2018_921_MOESM1_ESM.docx]

**Additional File 1: Full parameters table including distributions used in the model**

| Parameter | Mean (95% Confidence Interval) | Distribution | Reference |
| --- | --- | --- | --- |
| Base case parameters |  |  |  |
| Number of births per year | 400,308 | - | [1-3] |
| X-ALD incidence | 1 in 22,361 (15,083, 33,153) | Log-odds  Normal (-10.7,0.385) | [4-8] |
| Proportion  CCALD,AMN,Addison’s/Asymptomatic | 0.53 (0.36, 0.69), 0.32 (0.18, 0.49), 0.15 (0.05, 0.28) | Dirichlet (18,11,5) | [9] |
| Non-X-ALD peroxisomal incidence | 1 in 63,000 (33,897, 117,090) | Log-odds  Normal (-9.87, 0.254) | [8] |
| Age at presentation CCALD | 7 (6.76, 7.24) | Normal (7, 0.13) | [10] |
| Survival from onset CCALD  Weibull distribution  - shape parameter  - scale parameter  - correlation | -2.970  0.162  -0.8994 | Normal (-2.970,0.197)  Normal (0.162,0.067) | [10] |
| Time to CCALD progression (years) | 1.6 (1.34, 1.86) | Normal (1.6, 0.13) | [10] |
| Mortality risk HSCT | 0.08 (0.01, 0.21) | Beta (2, 23) | [11] |
| Proportion of CCALD currently undergoing early transplant (Family history) | 0.33 (0.23, 0.43) | Beta (28, 58) | [11] |
| Proportion ALD-DRS 0, ALD-DRS1, ALD-DRS2, ALD-DRS 3-4 after transplant | 0.62 (0.35, 0.85), 0.23 (0.05, 0.48), 0.08 (0.002, 0.26), 0.08 (0.002, 0.26) | Dirichlet (8,3,1,1) | [11] |
| Proportion successful HSCT develop AMN | 0.6 (0.19, 0.93) | Beta (3, 2) | [12] |
| Sensitivity | 0.995 | - |  |
| Specificity | 1 | - |  |
| Proportion of AMN mild | 0.51 (0.38, 0.64) | Beta (29,28) | [13] |
| Proportion of AMN developing adult onset cerebral X-ALD | 0.63 (0.44, 0.8) | Beta (17, 10) | [14] |
| Age at presentation AMN (years) | 35.3 (26.7, 43.9) | Normal (35.3, 4.39) | [14] |
| Time to development of adult onset cerebral X-ALD (years) | 10.2 (3.3, 17.1) | Normal (10.2, 3.52) | [14] |
| Survival adult onset cerebral X-ALD (years) | 3.4 (0.5, 6.3) | Normal (3.4, 1.48) | [14] |
| QALYs | See Additional File 2 |  |  |
| Costs | See Additional File 3 |  |  |
|  |  |  |  |

Reference List

1. Office for National Statistics. Birth Summary Tables - England and Wales 2014. 2015.<https://www.ons.gov.uk/peoplepopulationandcommunity/birthsdeathsandmarriages/livebirths/datasets/birthsummarytables>. Accessed 17^th^ November 2015

2. National Records of Scotland. Vital Events Reference Tables 2014. 2015. <https://www.nrscotland.gov.uk/statistics-and-data/statistics/statistics-by-theme/vital-events/general-publications/vital-events-reference-tables/2014>. Accessed 17^th^

3. Northern Ireland Statistics and Research Agency. Live births 1887 to 2014. 2015. <https://www.nisra.gov.uk/publications/live-births-1887-2015>. Accessed on 17^th^ November 2015

4. Kirk EP, Fletcher JM, Sharp P, Carey B, Poulos A. X-linked adrenoleukodystrophy: the Australasian experience. American Journal of Medical Genetics 1998;76:420-423.

5. Di Biase A, Salvati S, Avellino C, Cappa M, Bertini E, Moroni I et al. X-linked adrenoleukodystrophy: first report of the Italian Study Group. Italian Journal of Neurological Sciences 1998;19:315-319.

6. Bezman L, Moser AB, Raymond GV, Rinaldo P, Watkins PA, Smith KD et al. Adrenoleukodystrophy: incidence, new mutation rate, and results of extended family screening. Annals of Neurology 2001;49:512-517.

7. Jardim LB, da Silva AC, Blank D, Villanueva MM, Renck L, Costa ML et al. X-linked adrenoleukodystrophy: clinical course and minimal incidence in South Brazil. Brain & Development 2010;32:180-190.

8. Moser BA, Jones OR, Hubbard CW, Tortorelli S, Orsini JJ, Caggana M et al. Newborn Screening for X-Linked Adrenoleukodystrophy. International Journal of Neonatal Screening 2016,2:15

9. Horn MA, Retterstol L, Abdelnoor M, Skjeldal OH, Tallaksen CM. Adrenoleukodystrophy in Norway: high rate of de novo mutations and age-dependent penetrance. Pediatric Neurology 2013;48:212-219.

10. Mahmood A, Raymond GV, Dubey P, Peters C, Moser HW. Survival analysis of haematopoietic cell transplantation for childhood cerebral X-linked adrenoleukodystrophy: a comparison study. Lancet Neurology 2007;6:687-692.

11. Peters C, Charnas LR, Tan Y, Ziegler RS, Shapiro EG, DeFor T et al. Cerebral X-linked adrenoleukodystrophy: the international hematopoietic cell transplantation experience from 1982 to 1999. Blood 2004;104:881-888.

12. Van Geel BM, Poll-The BT, Verrips A, Boelens JJ, Kemp S, Engelen M. Hematopoietic cell transplantation does not prevent myelopathy in X-linked adrenoleukodystrophy: a retrospective study. Journal of Inherited Metabolic Disease 2015;38:359-361.

13. Keller JL, Wang JI, Kang JY, Hanson JA, Kamath P, Swain JO et al. Strength: a relevant link to functional performance in the neurodegenerative disease of adrenomyeloneuropathy. Neurorehabilitation & Neural Repair 2012;26:1080-1088

14. De Beer M, Engelen M, Van Geel BM. Frequent occurrence of cerebral demyelination in adrenomyeloneuropathy. Neurology 2014;83:2227-2231.
