# Supplementary material for: Economic impact of screening for X-linked Adrenoleukodystrophy within a newborn blood spot screening programme
Source: Orphanet J Rare Dis. 2018 Oct 11;13:179. doi: 10.1186/s13023-018-0921-4 (PMC6182830; doi:10.1186/s13023-018-0921-4)
Supplement: Supplementary file 2 — Systematic review of ALD for model parameters. This file contains the full methods and results of the systematic review that was carried out to inform the model parameters presented in this manuscript. (DOCX 315 kb) [file 13023_2018_921_MOESM2_ESM.docx]

**Additional File 2 Systematic review of ALD for model parameters**

Joanna Leaviss, Alice Bessey, James B Chilcott, Anthea Sutton.

Joanna Leaviss: j.leaviss@sheffield.ac.uk

Alice Bessey: [a.r.bessey@sheffield.ac.uk](mailto:a.r.bessey@sheffield.ac.uk)

James B Chilcott: j.b.chilcott@sheffield.ac.uk

Anthea Sutton: a.sutton@sheffield.ac.uk

School of Health and Related Research

The University of Sheffield

Regent Court

30 Regent Street

Sheffield

UNITED KINGDOM

S1 4DA

1. Review aims

Specifically the literature review aimed to provide data on the following modelling parameters:

1. Screening (sensitivity and specificity of newborn screening for X-ALD).
2. Incidence of X-ALD, including incidence of phenotypes (CCALD, AMN, Addison’s disease only, female X-ALD, other X-ALD). Available elsewhere, contact authors for details.
3. Morbidity and mortality (transplantation and no transplantation)
4. Costs (including screening, monitoring, transplantation, long-term costs post successful/unsuccessful transplantation).

2. Methods

2.1 Search Strategy

A comprehensive, systematic search of bibliographic databases was conducted to identify literature on X-ALD. To ensure a broad spectrum of evidence was retrieved at the outset, search techniques were aimed at maximising the rate of return of potentially relevant information. Search terms relating to X-ALD (including synonyms) were combined using Boolean Operators and database-specific syntax. All search strategies included free-text and thesaurus terms (where available). Searches were limited to humans and English language only. No study type or publication limits were applied. The following bibliographic databases were searched:

- MEDLINE & MEDLINE In-Process (via OvidSP)
- EMBASE (via OvidSP)
- CINAHL (via EBSCO)
- The Cochrane Library
- Web of Science Core Collection (Science Citation Index, Social Sciences Citation Index, Conference Proceedings Citation Index- Science, Conference Proceedings Citation Index- Social Science & Humanities)

All databases were searched from inception to present. Searches were conducted 12/03/2015-17/03/2015. The following search strategies were used:

MEDLINE

1 *Adrenoleukodystrophy/ (1231)

2 "x-linked ald".ti,ab. (34)

3 "x-linked adrenoleukodystrophy".ti,ab. (684)

4 adrenomyeloneuropathy.ti,ab. (343)

5 adrenoleukodystrophy.ti,ab. (1634)

6 1 or 2 or 3 or 4 or 5 (1862)

7 limit 6 to (english language and humans) (1532)

Embase

1 *adrenoleukodystrophy/ (1720)

2 "x-linked ald".ti,ab. (49)

3 "x-linked adrenoleukodystrophy".ti,ab. (881)

4 adrenomyeloneuropathy.ti,ab. (424)

5 adrenoleukodystrophy.ti,ab. (2010)

6 1 or 2 or 3 or 4 or 5 (2377)

7 limit 6 to (human and english language and embase and yr="2014 - 2015") (105)

The Cochrane Library

#1 MeSH descriptor: [Adrenoleukodystrophy] explode all trees

#2 x-linked ald

#3 x-linked adrenoleukodystrophy

#4 adrenomyeloneuropathy

#5 adrenoleukodystrophy

#6 #1 or #2 or #3 or #4 or #5

CINAHL

S1 (MM "Adrenoleukodystrophy")

S2 x-linked ald

S3 x-linked adrenoleukodystrophy

S4 adrenomyeloneuropathy

S5 adrenoleukodystrophy

S6 S1 OR S2 OR S3 OR S4 OR S5

Web of Science Core Collection

TI=((x-linked ald or x-linked adrenoleukodystrophy or adrenomyeloneuropathy or adrenoleukodystrophy))

Refined by: LANGUAGES: ( ENGLISH )

Indexes=SCI-EXPANDED, SSCI, CPCI-S, CPCI-SSH Timespan=All years

Additional search methods

Reference lists of key studies identified as potentially relevant to the model were checked for any further relevant references, and, if appropriate, the citation search facility in Web of Science was used to identify any relevant cited references and additional studies by key authors.

2.2 Review strategy

The purpose of the review was to populate model parameters, therefore the searches aimed to identify a diverse range of evidence. A number of types of pre-specified information were sought (outlined below) from a range of sources, however data searching and data extraction remained dynamic in order to reflect additional information needs identified during model development (Kaltenthaler et al. 2011 [1]). The following inclusion criteria were developed to identify studies containing data that would be considered for inclusion in the model.

2.2.1 Inclusion criteria

*Population*: Newborns, children or adults with X-ALD, including its phenotypes (CCALD, AMN, Addison’s disease, female X-ALD, other X-ALD). Studies including other inherited metabolic disorders/leukodystrophies were considered for inclusion if the N for X-ALD was not <5.

*Intervention*: Stem cell transplantation (bone marrow or cord blood); or no intervention (for natural history, incidence, screening and cost parameters).

*Study design*: Due to the diverse data requirements of the model, searches were not limited by study type, with the exception of case reports, which were excluded. Study designs considered included RCTs, non RCTs, observational studies (both longitudinal and cross-sectional). Studies where N<5 were excluded. Studies using animal models, narrative reviews, editorials, opinions, and non-English language papers were excluded. Conference abstracts were excluded where they contained insufficient data. Papers containing incidence data where data collection methods were not fully described were excluded from further consideration.

Comparator: N/A

*Outcomes*: Studies reporting any of the following outcomes were considered – incidence of X-ALD, including breakdown by phenotype; sensitivity and specificity of newborn screening; morbidity and mortality (natural history, i.e. with no treatment, or following treatment i.e. transplant success rate, survival rates, HRQoL, adverse events); economic costs (screening, confirmation costs, monitoring costs).

2.2.2 Quality assessment and examination of bias

The following steps were taken to minimise bias:

1. Quality assessment using a standardised tool appropriate for the study design was conducted for any study which was used to supply data to inform the model.
2. Where choices about values were needed, more than one team member was involved in the decision-making process. Team members included clinical advisors, information specialists, modellers, or systematic reviewers.
3. Due to the diverse nature of the information sources, a hierarchy of evidence was consulted when selecting data for the model (Coyle 2010).

2.2.3 Study selection

Titles and abstracts of all unique citations were screened independently by JL using the inclusion criteria above. Ambiguities were resolved by reference to AB. The full papers of all potentially relevant citations were retrieved for an in-depth assessment concerning inclusion.

2.2.4 Data extraction

Data relating to study characteristics of all full papers considered for inclusion in the model (study design, study populations, diagnostic criteria, details of interventions, outcome measures) were extracted by JL. Data selected for use in the model was double-checked from the original source by AB.

2.2.5 Data synthesis

Study characteristics for all studies considered at full paper stage for inclusion in the model will be summarised in a narrative synthesis. Where more than one study is identified that could provide data for a model parameter, and where heterogeneity between those studies is low, statistical synthesis of the evidence will be considered. Where synthesis is not deemed meaningful, values from the most relevant and appropriate study will be selected.

3. Results

3.1 Quantity of research identified.

2515 unique citations were retrieved by the searches. Search results broken down by source are presented in Table 1 below:

Table 1. Identified citations by bibliographic database.

| Database | Scoping Search | Full Searches | Total |
| --- | --- | --- | --- |
| Ovid MEDLINE 1946 to March Week 2 2015 | Approx 1,500 | 13 (from May 2014-present) | 1,533 |
| Embase 1974 to 2015 March 11 | Approx 1,500 | 110 (2014-present - month option not possible) | 1,563 |
| Cochrane Database of Systematic Reviews : Issue 3 of 12, March 2015 | N/A | 2 | 2 |
| Cochrane Central Register of Controlled Trials : Issue 2 of 12, February 2015 | N/A | 13 | 13 |
| Database of Abstracts of Reviews of Effect via The Cochrane Library : Issue 1 of 4, January 2015 | N/A | 0 | 0 |
| Health Technology Assessment Database via The Cochrane Library: Issue 1 of 4, January 2015 | N/A | 0 | 0 |
| NHS Economic Evaluation Database via The Cochrane Library: Issue 1 of 4, January 2015 | N/A | 0 | 0 |
| CINAHL via EBSCO 1960-present | N/A | 98 | 98 |
| Web of Science Core Collection Citation Indexes:  Science Citation Index Expanded (SCI-EXPANDED) --1900-present  Social Sciences Citation Index (SSCI) --1956-present  Conference Proceedings Citation Index- Science (CPCI-S) --1990-present  Conference Proceedings Citation Index- Social Science & Humanities (CPCI-SSH) --1990-present | N/A | 1, 208 | 1,208 |
| Total References Retrieved |  |  | 4,417 |
| Total Unique References |  |  | 2,515 |

After initial screening of titles and abstracts, 80 full papers were assessed in detail. 38 papers were subsequently excluded from further consideration (see appendix 1 for reasons for exclusions at full paper stage). Reasons for exclusion included case study (n=7); discussion/comment piece (n=2); conference abstract (n=3); intervention not stem cell transplant e.g. Lorenzo’s oil, Lovastin therapy, statin therapy (n=6); N<5 (n=2); no data e.g. overview of programme for patients with X-ALD (n=3); narrative review (n=3); MRI outcomes only not useful for model (n=5); attitudes towards screening (n=1); single symptom outcome (myelopathy) (n=1); mutation analysis/diagnostic test of carrier status (n=2); not prenatal or newborn screening (n=1); biomarker outcomes only (n=1); importance of diagnosis of adrenal insufficiency (n=1). 5 additional full papers were identified through citation searching or expert input. Therefore 47 papers remained eligible for further consideration for inclusion in the model. Figure 1 shows the PRISMA flowchart for the screening of retrieved citations.

The remaining studies were categorised into themes: screening (n=5); incidence (including frequency of phenotypes) (n=12); natural history (a study reporting morbidity and/or mortality data where no transplant is given) (n=12); intervention (a study reporting morbidity and/or mortality data following intervention (transplant) (n=14), costs (n=4). Where there was overlap, i.e. a study reported data for more than one theme, the study was categorised by its main theme.

Figure 1. PRISMA flowchart.

Studies excluded by title or abstract

(n=2435)

Potentially relevant studies identified and screened by title or abstract

(n=2515)

Studies excluded at full paper screen

(n=38)

Total full papers screened

(n=80)

Studies considered potentially relevant

(+ 5 papers from citation searching/expert input)

(n=47)

Screening n= 5

Incidence n=12

Natural history/progression n=12

Interventions n=14

Costs n=4

3.2 Quality Assessment:

Papers which supplied data for the model were assessed for quality using the CASP tool for cohort studies. Results are presented in appendix 2. All were assessed as good quality.

3.3 Results by parameter

3.3.1 Incidence

12 papers were retrieved that contained incidence data that was considered to be potentially relevant to the model. Table 2 shows summary characteristics for incidence studies. Of these, 9 studies calculated incidence rates, with 10 studies reporting frequency of phenotypes. Studies estimating incidence data were conducted in a number of studies – USA (Bezman 2001 [2]), Italy (Di Biase 1998 [3]), Germany (Heim 1997 [4]), Norway (Horn 2013 [5]), South Brazil (Jardim 2010 [6]), Australasia (Kirk 1998 [7]), France (Sereni 1993 [8]), Japan (Takemoto 2002 [9]), and the Netherlands (Van Geel 1994 [10]). No studies estimated incidence for the UK. The methods of estimating incidence varied between the studies. Bezman 2001 [2] estimated incidence based on the number of new diagnoses in two specialist Institutes over 2 different time periods (1981-1995 and 1996-1998). Diagnosis was by VLCFA levels, with samples also taken from at-risk relatives. Incidence estimates were calculated using live births in the USA for one year (1998). Several studies identified cases from one institution or laboratory (Jardim 2010 [6], Pereira 2012 [11], Ruiz 1998 [12], Kirk 1998 [7]), whilst others attempted to collect data from numerous hospitals or institutions within the study country through questionnaires or records review (Heim 1997 [4], Horn 2013 [5], Sereni 1993 [8], Takemoto 2002 [9], van Geel 1994 [10]). The superiority of either method is unclear, with response rates for some studies attempting to contact all possible institutions fairly low (e.g. Takemoto response rate 54%), whilst some single institution studies assumed that the majority of X-ALD cases for that country would be identified e.g. Kirk 1998 [7]. Diagnosis of cases was mainly reported as by elevated VLCFA levels, although some studies did not describe the method of diagnosis (Moser 1997 [13], Di Biase 1998 [3]) or simply reported ‘known patients’ (Takemoto 2002 [9]). Time periods covered for data collection ranged from the turn of the century (van Geel 1994 [10], range 1910-1990), the 1950s e.g. Horn 2013 [5], range 1956-1995, Takemoto 2002 [9], range 1950-1990, to more recently Jardim 2010 [6], range 2002-2006. Studies of earlier time periods may create bias as diagnostic methods were not as advanced (e.g development of VLCFA assays).

Estimated incidence rates for each study are presented in Table 3. These rates vary by study, and whilst some of this variation may be due to differences in data collection methods, as X-ALD is a genetic condition differences by country are not unexpected.

3.3.2 Frequency of phenotypes.

Ten studies report frequency of X-ALD phenotypes. Phenotypes include childhood cerebral (CCALD), adolescent cerebral, AMN, adult cerebral, asymptomatic X-ALD, Addison’s Disease only, or OPC. Definitions of each phenotype as described by each study are presented in Table 4. Frequency of each phenotype varies from study to study. Noticeably van Geel 1994 [10](Netherlands) report a high frequency of AMN with a lower frequency of cerebral cases as compared to other studies, whilst Sereni 1993 [8](France), Jardim 2010 [6](South Brazil) and Pereira 2012 [11](South America) report relatively low levels of AMN in comparison to other studies. These differences may represent variation in diagnostic methods, for example differentiating between AMN and asymptomatic cases at different stages of the condition. Figure 2 presents the frequency of different phenotypes as reported by the different studies.

Table 2: Summary characteristics for all incidence studies.

| **Study** | **Country** | **Study design** | **Method of diagnosis** |
| --- | --- | --- | --- |
| Bezman 2001 [2] | USA | Number of new male patients with X-ALD from the USA diagnosed at the Kennedy Krieger Institute between 1981 and 1995, and the combined total at the Institute and Mayo Clinic from 1996 to 1998. Estimates calculated from total number of live births in the USA in 1998. | Identification of probands by increased plasma VLCFA levels.  Samples from at-risk relatives on the basis of pedigree analysis. |
| Di Biase 1998 [3] | Italy | Incidence calculated using the number of diagnoses (57) and the number of males born (1,620,000) in the period 1990-1995. | N/R |
| Heim 1997 [4] | Germany | Epidemiological study of the leukodystrophies in Germany carried out in 1994. Data from 26 registries specialized in these disorders. | For ALD/AMN, diagnoses by biochemical assessment (elevation of VLCFAs in plasma, red blood cells, or cultured skin fibroblasts), or histological (multilamellar inclusions in brain tissue) criteria. |
| Horn 2013 [5] | Norway | Population-based cross-sectional point prevalence study. Cases of ALD identified by:  Searching records of diagnostic laboratories performing VLCFA or ABCD1 gene mutation analysis.  Letters to all departments of paediatrics, adult neurology and medical genetics in Norway.  Contact through experts. | Residents of Norway during 1945-2011. The population on prevalence day was 4,953,000 inhabitants.  Living subjects diagnosis through elevated VLCFAs or a disease-causing mutation in the ABCD1 gene. N=63, of which 39 (13 males, 26 females) were alive on prevalence day. |
| Jardim 2010 [6] | South Brazil | Males with X-ALD detected at 1 hospital in a 15 year period. Index cases were referred for genetic evaluation due to suspicion of ALD. | Measurement of VLCFAs and collection of family history. Classified into four phenotypes: CALD, AMN, Addison-only, asymptomatic |
| Kirk 1998 [7] | Australasia | Retrospective review of records collected over 15 year period at referral laboratory for the study of metabolic diseases. All known cases of ALD diagnosed in Australia or New Zealand between 1981 and 1996 and their families. Compared to combined annual birthrate for the same period of approximately 310,000. | Measurements of VLCFAs in plasma and cultured fibroblasts and genetic linkage studies. |
| Moser 1997 [13] | USA | Epidemiological survey of 826 kindreds – distribution of phenotypes calculated by 2 methods, 1) from 123 kindreds with 2 or more affected males (all hemizygotes n=637 and hemizygote proband excluded n=530); 2) from 253 sibships in which genotype and phenotype has been determined in all male members (all affected males n=388 and hemizygote proband excluded n=276). | Methods not described in the 1997 paper. |
| Pereira 2012 [11] | South America | Phenotype distribution identified through a molecular study of 38 unrelated families from South America. Mostly identified through the main study institution but others were ascertained through their physicians at other sites. 87 affected men were identified. | Patients previously diagnosed by VLCFA analysis. |
| Ruiz 1998 [12] | Spain | Phenotypes of X-ALD patients from regions of Spain diagnosed in 1 laboratory from 1988-1998. | Patients diagnosed by VLCFA measurements in serum, cultured skin fibroblasts, and mononuclear cells. Patients belonged to 48 kindred from a range of Spanish regions. |
| Sereni 1993 [8] | France | Retrospective review of records to identify cases for the whole of France. Response rate 80% for pediatric departments, 75% for neurologic and endocrinologic and 50% for neuropathic departments. | An elevation of VLCFAs, a histological observation of multilamellar inclusions in brain tissue, or a record of adrenal insufficiency and leukodystrophyic encephalopathy or paraparethesis in the same patient or the same family. |
| Takemoto 2002 [9] | Japan | Nationwide questionnaire survey sent to 4802 medical departments to identify patients with X-ALD. Follow-up questionnaires sent to those responding positively. 286 ALD patients identified between 1990 and 1999. The number of newly manifested patients was estimated to be about 20 per year in Japan, calculated from the 10 new patients reported every year and the 54% response rate of the survey. Because the number of male babies declined between the 1950s and 1990s in Japan from 1.0 million to 0.6 million per year, the incidence of X-linked ALD was estimated to be between 1:30000 and 1:50000 boys | Known patients. |
| Van Geel 1994 [10] | Netherlands | Search for as many X-ALD kindreds in the Dutch population as possible – between June and October 1992, departments of Neurology, Internal Medicine, Pediatrics and Clinical Genetics of Dutch hospitals with more than 200 beds were contacted. | Diagnosis by interview, physical examination and VLCFA assay. Further screening of relatives of identified X-ALD patients. |

Table 3: Summary of findings for incidence rates.

| **Study** | **Country** | **Years** | **Cases** | **Population size** | **Incidence** |
| --- | --- | --- | --- | --- | --- |
| Bezman 2001 [2] | USA | 1996-1998 | 96 | 2,016,205 | 1:21,000 male births |
| Di Biase 1998 [3] | Italy | 1990-1995 | 57 | 1,620,000 | 1:28,000 male births |
| Heim 1997 [4] | Germany | 1978-1994 | 188 males 70 females | N/R | 0.8:100,000 births |
| Horn 2013 [5] | Norway | 1956-1995 | 39 | 2,372,810 | 1.6:100,000 live births |
| Jardim 2010 [6] | South Brazil | 2002-2006 | 13 | 450,000 | 1:35,000 males |
| Kirk 1998 [7] | Australasia | 1981-1996 | 76 | 310,000 | 1.6:100,000 live births |
| Sereni 1993 [8] | France | 1956-1986 | 87 | N/R | 1:100,000 male births |
| Takemoto 2002 [9] | Japan | 1950-1990 | 286 | 1,000,000 declining to 600,000 | Between 1:30,000 and 1:50,000 males |
| Van Geel 1994 [10] | Netherlands | 1910-1990 | 77 | 8,000,000 | 1:100,000 male births |

Table 4: Definitions of phenotypes for studies reporting frequency of phenotypes.

| **Study** | **Definition of phenotypes** |
| --- | --- |
| Heim 1997 [4] | Definitions of phenotypes not given but based on ‘age at onset, presence of paraparesis, adrenal failure and leukodystrophic encephalopathy in MRI’. |
| Horn 2013 [5] | Definitions below reported in Horn – ‘modified after Moser 1997’.  CCER (childhood cerebral): progressive behavioural and cognitive neurological deficits, inflammatory brain demyelination.  Adol-CER (adolescent cerebral): Like CCER but onset in adolescence  Adult CER (adult cerebral): Rapid inflammatory cerebral demyelination resembling the childhood form, without preceding AMN.  AMN (adrenomyeloneuropathy): paraparesis progressive over decades, distal axonopathy, inflammation mild or absent, mainly spinal cord involvement.  AMN-CER (AMN-cerebral): onset like AMN, subsequent development of cerebral demyelination resembling childhood form.  ADD (Addison-only): primary adrenocortical failure without neurological abnormalities  ASYMP (asymptomatic): Harbouring the genetic condition of X-ALD without neurologic or endocrinologic abnormalities. |
| Jardim 2010 [6] | CALD: 10 clear encephalic signs: hemisyndromes (hemiparesias, hemihypoesthesias, hemianopsias), amaurosis, cognitive deterioration (aphasias, acalculias, agnosias, apraxias), axial and appendicular cerebellar syndromes and cranial nerve lesions, or 2) demyelinating lesions in neuroimaging exams of the encephalus.  AMN: If findings 1) and 2) were absent and if the following were present: 3) symmetric pyramidal signs, and/or 4) distal sensitive loss with bilateral symmetric and neuropathic pattern, and/or 5) neurophysiologic signs of peripheral neuropathy, or lesion of the ascending tract (in SSER or SSEP)  Addison-only: the absence of findings 1-5 in individuals with 6) frank or subclinical adrenal adrenal insufficiency (positive stimulation test with ACTH).  Asymptomatic: the absence of any of the findings 1-6. |
| Kirk 1998 [7] | Definitions of phenotypes ‘as per Moser 1995’. |
| Moser 1997 [13] | Childhood cerebral: Onset before 10 years of age, progressive behavioural and cognitive neurological deficits, inflammatory brain demyelination. Total disability often within 3 years.  Adolescent cerebral: Like childhood cerebral, but onset at 10-21 years of age.  AMN: Onset at 28+/- 9 years, paraparesis progressive over decades, distal axonopathy, inflammation mild or absent, mainly spinal cord involvement, cerebral involvement later in 45% of cases.  Adult cerebral: Rapid inflammatory cerebral progression resembling the childhood form, without preceding AMN, onset after 21 years of age.  Addison’s disease only: Primary adrenocortical insufficiency without neurological abnormalities.  Asymptomatic: ALD gene abnormality without neurological or endocrine abnormalities. |
| Pereira 2012 [11] | Definitions of phenotypes not given. |
| Ruiz 1998 [12] | Phenotypes defined using Moser 1992 definitions (age of onset, neurological signs, localization of brain lesions, adrenal impairment). |
| Sereni 1993 [8] | Childhood ALD (onset of symptoms before age 10 years)  Adolescent ALD (onset of symptoms between 10 and 21 years)  Adult cerebral ALD (onset of cerebral symptoms after age 21 years)  AMN (spinal cord and peripheral nerve involved mainly)  Addison’s disease without neurologic involvement  Pre-and asymptomatic |
| Takemoto 2002 [9] | CCALD: psychomotor regression between 2 and 10 years (mean age at onset: 7.1 years)  AMN: gait disturbance because of spinal cord involvement  Adult cerebral: characterized by dementia or psychological problems  OPC: gait disturbance and ataxia  Presymptomatic: no clinical manifestations  Addison’s disease: isolated insufficiency of the adrenal gland |
| Van Geel 1994 [10] | CCALD – normal psychomotor development. Onset of neurologic symptoms before 10 years. Behavioural disturbances, deterioration of vision, hearing, speech and gait, dementia. Often seizures and Addison’s disease. Vegetative state or death usually within 3 years after onset of neurologic symptoms.  Adolescent cerebral ALD – signs and symptoms as in CCALD, with onset between 10-21 years.  Adult cerebral ALD – dementia, schizophrenia, or other psychiatric disorders. Onset after 21 years. Symptoms of AMN may develop.  AMN – onset of neurologic symptoms most frequently in the third decade. Slowly progressive paraparesis. Often sensory disturbances of the legs, and impaired bowel and bladder function. Adrenocortical failure in most cases; often gonadal insufficiency (impotence, infertility).  Addison-only – adrenocortical insufficiency without neurologic symptoms. At risk for neurologic involvement.  Asymptomatic ALD – patients with the biological defect of X-ALD without the neurologic or endocrine abnormalities. |

Table 5: Summary of findings for frequency of phenotypes.

| **Study** | **Total cases** | **CCALD** | **Adol** | **CCALD or Adol** | **Cerebral** | **AMN** | **Adult cerebral** | **Asymptom** | **Addison only** | **OPC** |
| --- | --- | --- | --- | --- | --- | --- | --- | --- | --- | --- |
| Heim 1997 [4] | 188 males | N/R | N/R | N/R | N/R | 75 | N/R | N/R | N/R | N/R |
| Horn 2013 [5] | 34 | 14 | 4 | 18 | N/R | 11 | 0 | N/R | 5 | N/R |
| Jardim 2010 [6] | 51 | N/R | N/R | N/R | 27 | 11 | N/R | 8 | 5 | N/R |
| Kirk 1998 [7] | 95 | N/R | N/R | N/R | 51 | 24 | N/R | 5 | 15 | N/R |
| Moser 1997 a [13] | 637 | 235 | 44 | 279 | N/R | 203 | 19 | 45 | 83 | N/R |
| Moser 1997 b [13] | 530 | 191 | 27 | 218 | N/R | 170 | 16 | 48 | 80 | N/R |
| Moser 1997 c [13] | 388 | 151 | 23 | 174 | N/R | 101 | 8 | 50 | 54 | N/R |
| Moser 1997 d [13] | 276 | 91 | 11 | 102 | N/R | 72 | 6 | 50 | 47 | N/R |
| Pereira 2012 [11] | 87 | N/R | N/R | N/R | 54 | 14 | N/R | 6 | 6 | N/R |
| Ruiz 1998 [12] | 60 | 16 | 4 | 20 | N/R | 16 | 10 | 7 | 7 | N/R |
| Sereni 1993 [8] | 116 | 50 | 19 | 69 | N/R | 18 | 3 | 0 | 9 | N/R |
| Takemoto 2002 [9] | 154 | 46 | 14 | 60 | N/R | 39 | 33 | 7 | 0 | 13 |
| Van Geel 1994 [10] | 77 | N/R | N/R | 24 | N/R | 35 | 1 | 6 | 11 | N/R |

Figure 2: Frequency of phenotypes by study.

3.3.3 Screening:

Five studies were identified that reported data on the sensitivity or specificity of screening for X-ALD either pre-natally or at birth. Studies that described methods of screening beyond the post-natal period were not included, e.g. genetic testing for carriers. Table 6 shows summary characteristics for studies reporting data on sensitivity or specificity of the newborn or prenatal screening tests for X-ALD. One study(Lan 2010 [14]) reports data for a cohort study of pregnant women who had previously given birth to children with X-ALD. STR profiling of genomic DNA from amniotic fluid matched with samples of umbilical blood taken at birth. The remaining 4 studies reported data on the sensitivity or specificity of liquid chromatography tandem mass spectrometry in new-born blood spots (Hubbard 2006 [15], Hubbard 2009 [16], Theda 2014 [17], Turgeon 2015 [18]). All studies showed either 100% sensitivity or 100% specificity or both (Theda 2014 encountered 0 positives therefore no data is available for sensitivity). Turgeon 2015 demonstrated 100% identification of known ALD samples using a high throughput method [18]. No studies followed-up negative blood spots over time.

3.3.4 Natural History (progression/clinical course with no transplant):

12 studies were identified that provide data on the natural history/clinical course of patients with X-ALD who had not undergone transplant. Table 7 presents summary characteristics for all these studies. 7 studies provide data on the clinical course of patients with cerebral X-ALD (Mahmood 2007 [19], Suzuki 2005 [20], Liang 2004 [21], Moser 2000 [22], Pereira 2012 [11], Stephenson 2000 [23], Winstone 2015 [24]). 6 studies provide data on the clinical course of patients with AMN (De Beer 2014 [25], Keller 2012 [26], Pereira 2012 [11], Walterfang 2007 [27], Suzuki 2005 [20], Zachowski 2006 [28]). 1 study provides data on the clinical course of females with X-ALD (Engelen 2014 [29]). 4 studies provide data on patients with asymptomatic ALD (Kaga 2009 [30], Dubey 2005 [31], Cox 2006 [32], Pereira 2012 [11]). 1 study provides data on patients with Addison’s Disease only (Pereira 2012 [11]).

Table 6. Summary characteristics and outcomes for screening studies

| Study | Country | Study design | Population | Screening method | N | Results |
| --- | --- | --- | --- | --- | --- | --- |
| Hubbard 2006 [15] | USA | Laboratory analysis of blood spot samples | 25 male patients with X-ALD and 9 patients with peroxisome disorders compared to 19 controls | Combined liquid chromatography-Tandem mass spectrometry for X-ALD and other peroxisomal disorders. Blood spot area 0.0314 cm2 | N=19 normals (Mean age 0.39 years), X-ALD and AMN (Mean age 25.94 years) N=25, PBDs (Zellweger syndrome) (Mean age 4.69 years) N=9 | 100% sensitivity, 100% specificity |
| Hubbard 2009 [16] | USA | Laboratory analysis of blood spot samples | Newborn blood spots obtained from known individuals with X-ALD and peroxisomal disorders, and control anonymous newborn blood spots. | Combined liquid chromatography-tandem mass spectrometric method. Age of blood spot samples ranged from 2 to 13 years. | N=17 X-ALD or peroxisomal disorders, N=1000 masked control samples | Sensitivity of <1.0 fmole injected on colum with a correlation coefficient (R2) of 0.9987. All affected individuals were identified with one exception – one sample which was retrieved as affected did not have the biochemical or genetic abnormality of X-ALD and is therefore considered an error in sample identity. |
| Lan 2010 [14] | China | Cohort study of amniotic fluid | Pregnant women aged between 25 and 39 who had previously given birth to children with X-ALD. | Amniocentesis performed at 16-28 weeks of pregnancy. Isolation of genomic DNA, STR profiling. | N=12 fetuses. | 2 diagnosed as ALD males, 1 heterozygote, 4 normal unaffected females. Results of prenatal diagnosis match with samples of umbilical blood taken at time of delivery. |
| Theda 2014 [17] | USA | Laboratory analysis of dried bloodspots collected over a 21 month period. | Newborns at 3 Medical Centers/hospitals. | Liquid chromatography tandem mass spectrometry. | N=5000 newborn bloodspots collected. 293 labelled as insufficient to provide a punch for the study. N=4689 analysed. Results compared with n=16 X-ALD newborn bloodspots (non-study samples). | 0 positives encountered. Specificity 1.0. |
| Turgeon 2015 [18] | USA | Laboratory analysis of dried blood spots. | Patients with known X-ALD, peroxisomal biogenesis disorders and X-ALD carriers. Anonymised leftover NBS bloodspots. | High-throughput method for measurement of C20-C26 lysophosphatidylcholines and biochemical diagnosis of X-ALD. | X-ALD n=16, peroxisomal biogenesis disorders n=8, X-ALD carriers n=12. Total leftover bloodspots n=130. | 100% of known samples correctly identified. |

Table 7. Summary characteristics for natural history studies (no transplant)

| Study | Study design | Population | Diagnosis | N | Age | Outcomes (measures) | Results |
| --- | --- | --- | --- | --- | --- | --- | --- |
| Cox 2006 [32] | Cross-sectional study | Neurologically asymptomatic boys with normal brain MRI results and confirmed diagnosis of X-ALD | Diagnosis of X-ALD not reported | N=52 | Mean age: 6.7 +/- 3.6 years. | MRI results using Loes score (0.5 or less was classified as normal).  Neuropsychological function: Stanford-Binet Intelligence Scale, age appropriate Wechsler Intelligence Scale. | The study demonstrates an overall normal cognitive profile in asymptomatic boys with X-ALD who have no detectable abnormalities on conventional brain MRI.  All but 4 patients had normal cognitive function. |
| De Beer 2014 [25] | Prospective cohort study | Consecutive patients with AMN from the Dutch X-ALD cohort without cerebral demyelination on MRI at inclusion. | X-ALD confirmed biochemically by elevated levels of VLCFA or a mutation in the ABCD1 gene | N=27 | Mean age at inclusion = 39.0 =/- 8.7 years. Mean age at onset of AMN symptoms 35.3 +/- 8.6 years. | Cerebral demyelination, based on clear signs of spinal cord or peripheral nerve involvement, such as spastic paraparesis, sensory disturbances in the legs, and sphincter dysfunction. 3 levels of certainty – level A: definite cerebral demyelination, confirmed by MRI. Level B: probable cerebral demyelination with symptoms reported by physicians or relatives but no MRI possible. Level C: possible cerebral demyelination, suggested by signs and symptoms reported by family member or relatives. | 17/27 (63%): cerebral demyelination occurred between 25 and 66 years. Certainty was level A 10/17; level B 3/17; level C: 4/17. Mean survival after demonstration of cerebral demyelination was 3.4 +/- 2.9 years. |
| Dubey 2005 [31] | Prospective cohort study with follow-up until initiation of adrenal replacement therapy | Asymptomatic male patients with X-ALD | Identified through screening at-risk members of the family of known X-ALD patients. | N=49 | Mean age: 4.5 +/- 3.5 years. | Adrenal function (Serum adrenocorticotropic hormone ACTH and response to standard dose ACTH stimulation test). | At baseline, 39 (80%) of patients had some form of adrenal insufficiency. By the end of the study, 42 (86%) of patients had either biochemically definitive or borderline impairment of adrenal function. All patients showed normal brain MRI and neurologic exam. |
| Engelen 2014 [29] | Prospective cross-sectional cohort study | Female carriers of X-ALD over the age of 18 years. Recruited from outpatient clinics of the Academic Medical Centre and the Medical Centre Alkmaar from 2008-10. | Identified through known patients/carriers, confirmed through mutation analysis in 45 females. | N=46 | Age range 22 to 76 years (average 48 +/- 13 years). | Myelopathy, neuropathy, nerve conduction and electromyography, somatosensory evoked potentials, brainstem auditory evoked potentials. | Myelopathy: 29/46 (63%); peripheral neuropathy: 26/46 (57%); faecal incontinence: 13/46 (28%); frequency of symptomatic women increased with age (18% in women <40 years to 88% in women >60 years). |
| Kaga 2009 [30] | Prospective cross-sectional cohort study | Neurologically and radiologically asymptomatic CALD patients prior to HSCT | High serum VLCFA and/or abnormalities of the ABC gene | N=8 | Mean age 8.98 years +/- 3.97 years | Neuropsychological tests - IQ (age appropriate Wechsler Intelligence scale), K-ABC, visual perception (FDTVP), RCMT, RCFT. Visual evoked potentials. | Individual level reporting of outcomes. |
| Keller 2012 [26] | Cross-sectional cohort study | Analysis of baseline data from the first 142 participants with ALD enrolled in a trial of Lorenzo’s Oil. | Diagnosis confirmed by VLCFA assay and/or mutation analysis, with no brain demyelination, EDSS score 1 to 6.5. | N=142  60 males/82 females with AMN. | Mean age by stage of AMN:  Mild (male) 39 +/- 11 years; (female) 45 +/- 9 years.  Moderate (male) 41 +/- 12 years, (female) 53 +/- 12 years; severe (male) 39 +/- 15 years, (female) 50 +/- 4 years. | Body functions and structures, activity and participation. | Staging based on degrees of weakness reflects the continuum of disability. |
| Liang 2004 [21] | Retrospective cohort study, follow-up period 2 months to 8 years 5 months (mean 29.2 months). | Patients with a diagnosis of X-ALD in the National Taiwan University Hospital from 1993 to 2002 | Increased plasma VLCFA | N=9 | Mean age at diagnosis 7.4 years | Severity of neurologic involvement (Raymond scale); severity of MRI abnormality (Loes scale). | 3/9 patients died – 2 from complications of BMT, 1 from the disease 15 months after onset. 2/9 patients in vegetative state, 2/9 patients normal. 1/9 patients lost ability to communicate normally and had deteriorated neurological status. |
| Moser 2000 [22] | Prospective cohort study | Patients with X-ALD who had been referred to the Kennedy Krieger Institute during the preceding 12 years for treatment with Lorenzo’s Oil. | Diagnosis confirmed by clinical evaluation, MRI, plasma VLCFA assay and in 107 patients by mutation analysis. | N=372 | <3 years: 6.8%; 3-7 years: 17.1%; 7 to 10 years: 16%; 10 to 13 years: 12.5%; 13-16 years: 7%; 16 to 20 years: 5.4%; 20-24 years: 4.6%; 24-28 years: 5.1%; 28 to 32 years: 7.9%; 32-36 years: 5.1%; 36-40 years: 3.8%; > 40 years: 8.7% | Neurologic score (Raymond Scale), Neurological evaluations: language, visuospatial perception, visuomotor/graphomotor, memory and attention/executive function. Range of tests reported including age appropriate Wechsler Intelligence Scale, KABC, Stanford Binet, CVLT, KABC, TOVA.  Brain MRI | A range of Kaplan-Meier survival curves as a function of age and MRI abnormality at first contact. |
| Pereira 2012 [11] | Prospective cohort study | X-ALD patients and their families. Asymptomatic n=6, Addison only n=6, CALD n=54, AMN n=14, Unknown n=7. | VLCFA analysis | N=38 index cases, 30 males and 8 females. | Age at investigation (years): asymptomatic 6.4, Addison only 15.2, CALD 16.5, AMN 40.2. Age at onset (years): Addison only: 7.4, CALD 10.9, AMN 26.4. | Identification of genetic mutations, survival estimates. | Kaplan-Meyer curves on age at onset of each phenotype presented. |
| Stephenson 2000 [23] | Retrospective chart review | Patients with childhood onset X-ALD | Elevated VLCFA in the serum | 485, of which data reported for acute presentations (N=45/485 9.3%) | Overall mean age N/R. Average age for acute presentations = 5.5 years. | Outcomes reported for acute presentations only. Incidence of presentation symptoms as recorded on chart review. | 20/45 (44%) presented with seizure, of which 6/20 was facial seizure. 4/45 presented with generalized status epilepticus. 20/45 (44%) presented with adrenal crisis, at average age 5.2 years. 5/45 presented acutely with coma, mean age 5.3 years. |
| Suzuki 2005 [20] | Retrospective cohort study | 145 individuals with x-ALD: 46 cerebral form, 14 adolescent form, 13 OPC for. | N/R | N=145 | Mean year of appearance of each disturbance after onset. | Intellectual, visual, gait, sensory, psychic symptoms, and rate of progression (Kaplan-Meier plots) | Childhood: intellectual 0.2 (years), psychic 0.3, visual 0.5, hearing 1.3, gait 0.7, swallow 1.7, convulsion 1.3, adrenal 1.2. adolescent: intellectual 1.2, psychic 1.6, visual 1.1, hearing 1.9, gait 0.9, bladder 2.0, swallow 3.8, convulsion 2.6, adrenal 1.0. AMN: intellectual 14.0, psychic 10.2, visual 16.9, hearing 14.0, gait 0.0, sensory 3.2, bladder 8.4, swallow 13.0, adrenal 7.5. Adult cerebral: 1.5, psychic 10.2, visual 1.2, hearing 3.0, gait 1.8, sensory 1.9, bladder 2.3, swallow 4.0, adrenal 4.1. OPC: intellectual 2.0, psychic 2.1, gait 0.1, sensory 0.7, bladder 2.1, swallow 2.1, adrenal 2.7. |
| Walterfang 2007 [27] | Cross-sectional cohort study | Individuals with AMN. Presenting diagnosis 4/10 Addison’s disease, 6/10 AMN. | Genetic testing | N=10  9 males, 1 manifesting female carrier. | Median age 49.50 (range 27.25-56.25) years. | Neurological and functional status (clinical examination and the modified BI). Psychiatric symptoms (Structured Clinical Interview, self-report through BDI, BAI, clinician-rated through BPRS and SF-36). Cognitive screening (Mini Mental State Examination MMSE and NUCOG. | 2/10 had evidence of an affective illness, 5/10 had evidence of a lifetime history of affective illness. MMSE score ranged from 29-30. Median NUCOG score was 94 (indicates normal). Median BDI score 12 (range 6.25-24.25), indicative of low levels of anxiety. Median BPRS score 33 (range 29.25-37.75), indicating very mild psychiatric symptoms. Median SF-36 score 97.50 (range 87.00-104.25) (did not reach the threshold for caseness). Median BI score 100 (range 87.50-100) indicating low level of functional impairment. Median EDSS score 2.50 (range 1.375-6.00), indicating generally ambulatory with some level of physical disability. Adrenal state did not influence measures of cognition. |
| Winstone 2015 [24] | Prospective cross-sectional study | Boys with ALD. | Presenting symptoms: 42/55 deterioration in intellect and/or behaviour; 8/55 acutely (3 vomiting, 1 recurrent infection, 1 febrile encephalopathy, 1 hemi-seizures, 1 collapse, 1 acutely); 1/55 abnormal gait, 1/55 delayed development; 3/55 positive family history. Diagnosis confirmed by genetic study in 20/55. | N=74  55/74 were symptomatic (neurological symptoms) | Age at presentation 2-15 years 4 months (median 6 years) | Neurological and functional status | Of those symptomatic: 21/55 visual disturbances; 11/55 developed seizures; 28/55 had CT scans of which 8/28 showed leukoencephalopathy, 8/28 were normal, 3/28 showed cerebral infarction, 3/28 non-specific abnormalities. 54/55 had MRI scans of which 47/54 showed a leukoencephalopathy, 2/54 normal, 1/54 showed an infarct. VLCFAs were abnormal in 52/55. 37/53 had abnormal synacthen tests, 3/53 normal, 13/53 not available. |
| Zachowski 2006 [28] | Cross-sectional cohort study | Men with AMN and healthy controls | VLCFAs assay and mutation analysis | 20 AMN | AMN group mean age 34.3 +/_ 2.6 years. | Overall AMN severity (EDSS); Sensory, strength, and spasticity impairments. Subgroups based on severity of impairments: no loss (no significant mobility deficits); sensory loss (sensory loss only); sensory and strength loss; sensory and strength loss with spasticity. | Individuals with sensory loss, concomitant strength and sensory loss resulted in slower walking, with abnormal knee control; increased spasticity led to an exaggerated trunk motion and a knee-flexed posture. Hip strength was an independent predictor of walking velocity in AMN subjects. NB more similar results on sensorimotor abnormalities. |

Tables 8-12 present a narrow summary of key data for each of these studies considered most pertinent to the selection of data for the model. Tables are divided by phenotype. Where studies report data for more than one phenotype, the study appears in each relevant table.

Table 8. Cerebral

| Paper | Study design | N | Age | Duration of follow-up | Outcomes | Survival curves? |
| --- | --- | --- | --- | --- | --- | --- |
| Liang 2004 [21] | Retrospective chart review | 9, of which 6 not transplanted. | Mean age at diagnosis 7.4 years (range 2 years 9 months-13 years). | Follow-up periods range 2 months to 8 years 5 months. | Mortality. Neurological symptoms. | No |
| Mahmood 2007 [19] | Retrospective survival analysis | 283 | Mean age at onset of symptoms 7 years (SD 2 years) | Mean follow-up period 5.9 years | Mortality (5-year and 10- year survival from baseline MRI), adrenal function, neurological deficit. | Yes |
| Moser 2000 [22] | Prospective cohort study | 377 | Mean age N/R. Age at first contact <16 years. | Mean observation period 38 months | Severity of brain MRI involvement (Loes score) | No |
| Pereira 2012 [11] | Prospective cohort study | 54 CCALD (27 already deceased) | Mean age at onset 10.9 years (range 9.1-12.7); mean age at study 16.5 years (range 13.9-19) | Study period 6 years | Mortality | Yes |
| Stephenson 2000 [23] | Retrospective chart review | 485 of which 45 presented acutely | Mean age at acute presentation 5.5 years | No follow-up | Nature of presenting symptoms: seizures, adrenal crisis, encephalopathy or coma. | No |
| Suzuki 2005 [20] | Retrospective questionnaire study | 145 of which 46 CCALD, 33 adult cerebral, 14 adolALD, 13 OPC (and 39 AMN). | Mean age at onset CCALD < 8 years: 5.7 years; >8 years: 8.9 years; Adolescent 14.6 years; AMN 30 years; OPC: 34.8-39.3 depending on cerebral involvement. | Observation period >15 years | Disturbances of gait, intellectual, psychic, swallow, visual, hearing, sensory, bladder, convulsion, adrenal. | Yes |
| Winstone 2015 [24] | Prospective study of symptoms of ALD at presentation | 74, of which 55 symptomatic, 19 asymptomatic | Median age at presentation (symptomatic) 6 years (range 2-15 years) | No follow-up | Progressive intellectual and neurological deterioration. | No |

Table 9. AMN

| Paper | Study design | N | Age | Duration of follow-up | Outcomes | Survival curves? |
| --- | --- | --- | --- | --- | --- | --- |
| De Beer 2014 [25] | Prospective cohort study | 27 | Mean age at onset of symptoms 35.3 +/- 8.6 years; mean age at study 39.0 +/- 8.7 years. | Mean follow-up 16.8 +/- 2.1 years (alive); 9.9 +/- 5.2 years (dead). | Cerebral demyelination, mortality | No |
| Keller 2012 [26] | Cross-sectional cohort study | 142 | Mean age at study men: 40 +/- 12 years; women: 47 +/- 11 years | No follow-up | Body functions and structures, activity and participation by stages mild, moderate, severe. | No |
| Pereira 2012 [11] | Cohort study | 14 AMN (3 already deceased) | Mean age at onset 26.4 years (range 20.3-32.5); mean age at study 40.2 years (range 32.5-47.8) | Study period 6 years | Mortality | Yes |
| Suzuki 2005 [20] | Retrospective questionnaire study | 39 | Mean age at onset: With cerebral involvement 30.8 years, without cerebral involvement 30.0 years. | Observation period >15 years | Disturbances of gait, intellectual, psychic, swallow, visual, hearing, sensory, bladder, convulsion, adrenal. | Yes |
| Walterfang 2007 [27] | Cross-sectional cohort study | 10 | Median age at study 49.50 (range 27.25-56.25) years. | No follow-up | Psychiatric, cognitive and disability variables. | No |
| Zachowski 2006 [28] | Cross-sectional cohort study | 20 | Mean age at study 34.3 +/- 2.6 years | No follow-up | Sensorimotor function and axonal integrity | No |

Table 10. Female carriers

| Paper | Study design | N | Age | Duration of follow-up | Outcomes | Survival curves? |
| --- | --- | --- | --- | --- | --- | --- |
| Engelen 2014 [29] | Prospective cross-sectional cohort study | 46 | Age at study 48 +/- 13 years, range 22 to 76 years. | No follow-up | Myelopathy, peripheral neuropathy, faecal incontinence. | No |

Table 11. Asymptomatic ALD

| Paper | Study design | N | Age | Duration of follow-up | Outcomes | Survival curves? |
| --- | --- | --- | --- | --- | --- | --- |
| Cox 2006 [32] | Cross-sectional | 52 | Mean age at study 6.7 +/- 3.6 years, median 6.2 years, range 2.1 to 14.6 years. | No follow-up | Neuropsychological functioning (IQ, language, visuospatial skills, perception, visuomotor or graphomotor skills, memory, attention or executive function), adaptive skills, academic achievement. | No |
| Dubey 2005 [31] | Prospective cohort study | 49 | Age at study 4.5 +/- 3.5 years. | Mean follow-up 2 +/- 1.7 years | Adrenal insufficiency (ACTH test) | No |
| Kaga 2009 [30] | Prospective cross-sectional cohort study | 8 | Mean age at study 8.98 +/- 3.97 years | No follow-up | Neuropsychological functioning, visual evoked potentials, | No |
| Pereira 2012 [11] | Cohort study | 6 asymptomatic | Mean age at study 6.4 years (range 1.9-10.8) | Study period 6 years | Mortality | Yes |

Table 12. Addison’s Disease only

| Paper | Study design | N | Age | Duration of follow-up | Outcomes | Survival curves? |
| --- | --- | --- | --- | --- | --- | --- |
| Pereira 2012 [11] | Cohort study | 6 Addison’s only | Mean age at onset 7.4 years (range 5.4-9.4); mean age at study 15.2 years (range 5.3-25) | Study period 6 years | Mortality | Yes |

*Cerebral*: 7 studies reported natural history data for cerebral XALD. 4 studies were retrospective, reviewing medical records or administering questionnaires (Mahmood 2007 [19], Suzuki 2005 [20], Liang 2004 [21], Stephenson 2000 [23]), with 3 studies using a prospective design (Moser 2000 [22], Pereira 2012 [11], Winstone 2015 [24]). 2 studies did not report follow-up of patients (Stephenson 2000 [23], Winstone 2015 [24]), with the remaining studies reporting mean observation periods of 3 to 15 years. The key outcomes studied were mortality and severity of neurologic symptoms.

*AMN*: 6 studies reported natural history data for AMN. 3 studies of patients with AMN were cross-sectional with no follow-up reported (Keller 2012 [26], Walterfang 2007 [27], Zachowski 2006 [28]). The remaining studies had follow-up periods of between 6 and 15 years. Outcomes of interest were mortality, neurologic and psychological functioning, cerebral demyelination.

*Female carriers*: One study (Engelen 2014 [29]) focused on female carriers of X-ALD. This prospective cross-sectional cohort study had no follow-up. Outcomes of interest were myelopathy, peripheral neuropathy and faecal incontinence.

*Asymptomatic*: 4 studies reported data on asymptomatic patients. 2 studies recorded follow-up data of 2 and 6 years (Dubey 2005 [31], Pereira 2012 [11]), whilst 2 studies did not report follow-up data (Kaga 2009 [30], Cox 2006 [32]). Outcomes of interest were neuropsychological functioning, adrenal insufficiency, and mortality.

*Addison’s only*: 1 study reported data on patients with Addison’s Disease only (Pereira 2012 [11]). 6 Addison’s patients were observed for 6 years as part of a wider study of a patients with a range of phenotypes. Mortality rates were recorded.

3.3.5 Intervention (transplant):

14 studies reported data on a total of 266 patients with X-ALD who underwent transplant. Mahmood 2007 [19] and Gess 2008 [33]present follow-up data from Peters 2004 [34]. Several studies were not exclusively of transplant in X-ALD patients. Sakata 2004 [35]studied a population of children with congenital genetic diseases, of which 7 had X-ALD. Martin 2006 [36]studied a population of children with lysomal disorders, of which 6 had X-ALD. Gassas 2011 [37]studied a population of children with IMGDs, of which 6 had X-ALD. Prasad 2008 [38] also studied a population of children with IMGDs, of which 13 had X-ALD. Tables 13 and 14 present summary characteristics and outcome data for all studies identified as having potential to supply data on the clinical course of X-ALD following transplant.

A minority of studies recorded reasons for diagnosis of X-ALD. Baumann 2003 [39], Gess 2008 [33], Peters 2004 [34], and Miller 2011 [40] all report diagnosis of X-ALD for both symptomatic and non-symptomatic individuals. Diagnosis was either through signs and symptoms or, where non-symptomatic, through screening where there was known family history. Diagnosis of patients was through elevated plasma concentrations of VLCFA in all studies where method of diagnosis was reported. Most studies also reported diagnosis through evidence of cerebral disease confirmed by a positive MRI scan. Neurologic/neuropsychological assessments were reported in several studies (Baumann 2003 [39], Beam 2007 [41], Loes 1994 [42], Mahmood 2007 [19], Miller 2011 [40], Peters 2004 [34], Shapiro 2000 [43]). Prasad 2008 [38]report confirmation of diagnosis through DNA mutation analysis where possible.

All transplants were carried out at less than 19 years of age, with age ranges 5-13 years (Baumann 2003 [39]), 5.3-13.3 years (Gassas 2011 [37]), 6-16 years (Gess 2008 [33]), 3 months-11 years 9 months (Loes 1994 [42]), 4.9-18.6 years (Peters 2004 [34]), or median ages 7.1 years (Beam 2007 [41]), mean age 8.7 years (Miller 2011 [40]), 7.9 and 1.6 years (abnormal and normal adrenal function) (Petryk 2012 [44]). Study interventions were transplant of either bone marrow or peripheral blood stem cells. Some studies used both related and unrelated donors (Gess 2008 [33], Miller 2011 [40], Peters 2004 [34], Petryk 2012 [44], Shapiro 2000 [43]), whilst others reported unmatched transplantation only (Beam 2007 [41], Martin 2006 [36], Prasad 2008 [38], Sakata 2004 [35]). Conditioning/preparative regimens were described in the majority of studies.

Outcomes recorded were mortality, estimated survival probabilities, MRI severity, and a range of neurologic and neuropsychological functioning, e.g. IQ, vision, hearing, speech, gait, fine motor skills. Follow-up periods ranged between 180 days post-transplantation (Martin 2006 [36]), to 10 years and beyond (Shapiro 2000 [43], Prasad 2008 [38], Peters 2004 [34], Mahmood 2007 [19]). Key findings from the studies were that prognosis after transplantation is affected by the severity of neurologic symptoms/MRI severity pre-transplant, with greater severity and/or number of symptoms associated with poorer outcomes (Baumann 2003 [39], Gess 2008 [33], Mahmood 2007 [19], Miller 2011 [40], Peters 2004 [34], Wilken 2003 [45]).In addition, individual studies reported moderate correlation between age of onset and 5 year survival (Mahmood 2007 [19]), and no difference in survival in patients previously treated with Lorenzo’s Oil (Mahmood 2007 [19]). Adrenal insufficiency did not appear to show improvement after transplant (Petryk 2012 [44], Shapiro 2000). Where reported, causes of deaths post-transplant included disease progression, graft failure, infection, haemorrhage, aGVHD, haemolytic anaemia, severe VOD (Peters 2004 [34], Miller 2011 [40], Wilken 2003 [45], Beam 2007 [41], Baumann 2003 [39]).

4. Discussion and limitations of review:

The aim of the review was to identify data to inform modelling parameters used to provide an assessment of the cost-effectiveness of newborn screening for X-ALD. In particular, data was sought on the sensitivity and/or specificity of newborn screening, the incidence of X-ALD and the frequency of phenotypes, and the morbidity and mortality associated with X-ALD, both with and without transplant. The need for this broad spectrum of information was reflected in the search strategy, which aimed to maximise the rate of return of potentially relevant information. As a result, a large quantity of papers were retrieved (N=2515), which, after citation screening of relevant papers, yielded 47 papers from which data with potential to be included in the model was extracted.

Despite this large number of studies, no data was identified giving incidence of X-ALD or frequency of phenotypes in the UK. Since the review was conducted the progressive intellectual and neurological deterioration (PIND) in children study has published a lifetime risk of 6.6 cases of X-ALD per 1 million births. This is based on patients being reported via the British Paediatric Surveillance Unit. However, this is likely to underestimate the true incidence of X-ALD in the UK as the study only included patients under the age of 16 and who met the criteria [46]. Studies set in other countries showed variation in incidence rates. Differences may be due to methods of data collection (e.g. sampling from limited institutions, dates of data collection pre/post development of VCLFA assays), or due to genetic variation within populations. Differences in reported frequencies of phenotypes may be due to variation in diagnostic criteria used or advances in diagnostic techniques. More recent studies reporting frequencies of phenotypes may be more accurate, for example in identifying cases of AMN.

5 screening studies were identified. 1 of these studies reported sensitivity and specificity of pre-natal DNA profiling from amniotic fluid. The remaining 4 studies tested the sensitivity and specificity of combined liquid chromatography-tandem mass spectrometry in newborn bloodspot samples. Whilst none of these studies showed less than 100% sensitivity or 100% specificity, negative results have yet to be followed-up long-term.

26 studies were identified as providing potentially useful data on mortality and morbidity. 14 of these reported data on patients with X-ALD who underwent bone marrow or peripheral blood stem cell transplant. 12 studies reported data on the natural history of the disease without transplant. Key findings from the transplant studies indicate that prognosis is related to severity of the disease at the time of transplant, with poorer outcomes reported in patients with cerebral involvement, i.e. greater number or more severe neurologic/neuropsychological symptoms. Non-transplant studies reported indications of the natural history of the ALD without transplant. Prognosis varied by phenotype. Without transplant, outcomes for patients with cerebral involvement were generally shown to be poor, with better outcomes for those without cerebral involvement.

Whilst a broad approach to searches maximised retrieval of unique citations, there is still a possibility of missed data. Multiple parameters of interest present difficulties when sifting large volumes of titles and abstracts, which can result in exclusion of studies that may, on inspection of the full paper, report data that may be relevant to the model and therefore should be considered for inclusion. For example, intervention studies where the intervention was not transplant (e.g. Lorenzo’s oil, Lovastatin therapy) were excluded at sifting stage, however these may have contained data on natural history from control groups, or frequencies of phenotypes. Citation searching of included studies was conducted in an attempt to minimise this risk. Double-sifting by another reviewer would have minimised the risk further if time and resources would have allowed. Conference abstracts were excluded at sifting stage, and further searching of these, unpublished and grey literature may also yield UK data on incidence which is currently lacking.

Table 13. Summary characteristics for transplant studies.

| Study | Study design | Follow-up | Indications for transplantation | N total | % (N)  Symptomatic/  Asymptomatic or reason for diagnosis | Mean age |
| --- | --- | --- | --- | --- | --- | --- |
| Baumann 2003 [39] | Cohort study, patients with X-ALD undergoing HSCT | Patients who survived 1^st^ year followed up. Follow-up period 1.9 to 5.5 years. | MRI changes with signs of progressive disease such as expanding lesions, characteristic gadolinium enhancement and in addition on the development of neuropsychological impairment. Following rapid post-transplant deterioration of 3 patients who showed extensive MRI changes and moderate to marked clinical symptoms, criteria for subsequent patients were modified to exclude advanced stage of disease. Only no, mild or moderate stage included. | N= 14, 2 died from transplant-related complications, N=12 analysed | 9 patients showed signs of cerebral demyelination on MRI at referral.  5 patients detected by extended family screening had no cerebral involvement at diagnosis. These patients were prospectively evaluated and HSCT performed when signs of X-ALD appeared. | Age at transplantation range 5-13 years. |
| Beam 2007 [41] | Cohort study, patients with X-ALD lacking HLA-matched related donors undergoing HSCT | Median follow-up 3.3 years (range 12 days to 6.3 years). | N/R | N=12. 1 boy died whilst receiving chemotherapy but before transplant. | N/R | Median age at diagnosis 7 years (neonate to 9.75 years). Median age at initiation of chemotherapy 7.1 years. |
| Gassas 2011 [37] | Cohort study of patients with IMGDs who received allogenic HSCT in the Hospital for Sick Children. includes small sample with ALD | 3 monthly follow-up for the first 2 years and 6 monthly follow-up thereafter. | N/R | Total N=45 HSCTs for 44 children with IMGDs. N=6 children with X-ALD | N/R | HSCT median age 7.4 years (range 5.3 to 13.3 years). |
| Gess 2008 [33] | Retrospective chart review of patients treated with HCT for X-ALD | Follow-up of subset of Peters et al 2004 (N=94), who had an eye examination before and after receiving HCT (n=14). Patients included if they survived at least 100 days after HCT. Median follow-up not reported. | All boys had increased concentrations of fasting plasma VLCFA and had evidence of cerebral disease, as confirmed by MRI. | N=14 | 9/14 showed disease manifestation. 5/14 diagnosed through family member diagnosis. | Median age at transplant 9.5 years (range 6.0-16 years) |
| Loes 1994 [42] | Cohort study of 8 boys with CCALD who underwent successful bone marrow transplantation. | Mean MR follow-up 19 months (range 12 to 24 months). | N/R | N=8, 1 failed to return for follow-up and subsequently died. Data reported for 7 patients. | N/R | Age range at time of transplantation 5 years 3 months – 11 years 9 months. Mean age 8 years 10 months. |
| Mahmood 2007 [19] | Retrospective survival analyses on 283 patients with CCALD who had not received HCT with severity scores matched with 19 patients who underwent HCT from Peters et al 2004. | Follow-up of Peters et al 2004. Patients followed-up for 5.9 years (5.3, range 1 month to 30 years). | N/R | N=283 patients with CCALD or adolescent cerebral form of ALD who had not received HCT. 131 (46%) patients died within follow-up period N=30 patients matched for neurological disability and MRI severity scores with N=19 transplanted subgroup from Peters et al 2004. |  | Mean age of onset of symptoms 7 years (SD 2.1). Mean age at death of patients who dies during follow-up period = 12.3 years (4.9). |
| Martin 2006 [36] | Cohort study – Cord Blood Transplantation Study (COBLT) – unrelated donor umbilical cord blood transplantation in pediatric patients with lysosomal and peroxisomal storage diseases. | 180 days after transplantation. | Patients diagnosed with LSD whose developmental quotient, IQ, or clinical neurodevelopment examination demonstrated a level of functioning at which continuous life support would not be predicted to be required in the year after transplantation were eligible for enrolment. | Total N = 69 patients with lysosomal disorders. ALD n=8. | N/R | Mean age not given separately for ALD. |
| Miller 2011 [40] | Cohort study of 60 boys with CCALD undergoing HCT | Median post HCT follow-up 3.7 years. | A diagnosis of ALD based on abnormal plasma VLCFA profile findings and the presence of active cerebral disease evidenced by characteristic white matter signal changes on brain MRI. | N=60 | Reason for diagnosis 17/60 (28%) family history, 37/60 (62%) signs/symptoms, unknown 6/60 (10%). NFS at HCT 0 23/60 (38%), 1 17/60 (29%), >2 20/60 (33%). Loes score <10 30/60 (50%), >10 30/60 (50%). Adrenal insufficiency before HCT yes 43/60 (72%), no 10/60 (17%), unknown 7/60 (12%). | Mean age at HCT 8.7 years |
| Peters 2004 [34] | Multi-centre retrospective cohort study of 126 boys with X-ALD who underwent HCT. | Median 3.1 years post HCT (range 0.4-11.2 years). | Patients with X-ALD. | N=94 | Reason for diagnosis 28/94 (33%) family history, 58/94 (67% signs and symptoms, unknown 8/94. | <19 years of age, median age at HCT 9.0 years (range 4.9 to 18.6). |
| Petryk 2012 [44] | Retrospective chart review of 50 consecutive patients with ALD who had survived more than one year after HCT. | 1-7 years post HCT. | Patients with cerebral ALD | N=20 | N/R | Mean age at HCT by adrenal function group (pre/post HCT): abnormal/abnormal n=16 7.9 +/- 2.3 years; normal/abnormal n=3 1.6 +/- 0.6 years; normal/normal n=1 4 years. |
| Prasad 2008 [38] | Consecutive cohort study of children with a range of inherited metabolic disorders undergoing transplantation with partially HLA-mismatched unrelated donor umbilical cord blood. | Median follow-up 4.2 years (range 1-11 years). | Patients with inherited metabolic disorders. | Total N=159, ALD n=13 | N/R | No individual data for ALD. |
| Sakata 2004 [35] | Retrospective analysis of 81 patients with congenital genetic diseases who were treated with bone marrow transplantation from unrelated donors | N/R | Patients with congenital genetic diseases. | Total N=81, ALD n=7 | N/R | No individual data for ALD |
| Shapiro 2000 [43] | Cohort study with long-term follow-up of patients with X-ALD who underwent bone marrow transplantation. | 5-10 year follow-up | All patients showed typical abnormalities of cerebral X-ALD | Total N=12 | N/R | Mean age N/R. Ages at diagnosis reported for individual patients. |
| Wilken 2003 [45] | Cohort study with follow-up | Up to 5 years follow-up | If disease progression towards cALD was diagnosed based on neurologic, neuropsychologic, MRI, and MRS findings, HSCT was performed as soon as possible. | N=12 | N/R | Mean age N/R. ‘Most patients had ages of onset well below 10 years’. |

Table 14. Outcomes data for transplant studies.

| Study | Diagnosis | Intervention | Outcomes/measure |
| --- | --- | --- | --- |
| Baumann 2003 [39] | Diagnosis by elevated plasma concentrations of VLCFA and symptoms or elevated VLCFA and family relation in asymptomatic patients.  Neurological evaluation by modified scale developed by X-ALD-International Research Group (Kohler & Sokolowski).  Standardised neuropsychological tests including Wechlser Intelligence Scales, visual perception, visual memory, graphomotor, motor and speed, auditory short term memory, verbal memory, arithmetic. | HSCT. 10 patients with bone marrow, 2 patients with peripheral blood stem cells.  Preparative regimen: busuphan and cyclophosphamide, combined with antithymocyte or antilymphocyte globulin in 10 patients. | Neurological outcomes, neuropsychological outcomes, MRI scans (Loes score). 6 patients showed moderate to severe clinical deterioration after HSCT including 2 who died within 6 months. In this group, a MRI severity score of 10 or higher before HSCT was associated with severe impairment and a score of more than 12 was followed by rapid deterioration and death after HSCT. 6 patients showed no deterioration in neurological or neuropsychological assessment after HSCT. The presence of neurological symptoms affected prognosis, although individual patients showed an unexpected course. |
| Beam 2007 [41] | Baseline brain MRI, peripheral nerve conduction velocity, brainstem auditory evoked responses, visual evoked potentials, electroencephalogram, neurodevelopmental evaluations. Diagnosis confirmed by presence of abnormally high levels of long chain fatty acids in the blood. | Unrelated cord blood transplantation.  Conditioning regimen: busulfan, cyclophosphamide, antithymocyte globulin. | OS at 6.25 months was 66.7% (95% CI 39.9-93.3%). No events after this time point. Posttransplant, 2 patients died of progressive ALD, 1 patient died of severe VOD. 1 patient experienced primary graft failure, but engrafted after a second UCB transplant. 5/11 evaluable patients developed grade 1 GVHD, 2 developed grade 2, 2 developed grade 4 GVHD. cGVHD occurred in 2 patients.  NB range of data for individual neurological and neuropsychology outcomes available. |
| Gassas 2011 [37] | N/R | Allogenic HSCT. No individual breakdown of type for ALD patients.  Conditioning regimen: GVHD prophylaxis dependent on bone marrow or cord stem cell transplant. | Demyelination pattern from MRI available for 5/6 patients. 3/5 had posterior corpus callosum white matter changes extending into both hemispheres. 1/5 had changes only in splenium. 1/5 had more diffuse demyelination. 2/6 suffered secondary graft loss and died from progressive disease. 4/6 are long term survivors. All are completely ambulatory and independent in daily living activities. |
| Gess 2008 [33] | Diagnosis through increased VLCFA and evidence of cerebral disease confirmed with MRI. Demyelination pattern was parieto-occipital (n=9) or frontal (n=5). Median MRI severity score before HCT was 9.5 (range 6-16) (?Exact same as age -check error in journal). Median performance IQ score pre-HCT was 76 (range 46-131). The median visual acuity loss post-HCT was 0.36 log/MAR units (the equivalent of a change from 20/20 to 20/45) (range 0-1.90). | Related HCT n=2, unrelated n=8 marrow, n=4 cord blood).  Conditioning regimen: cyclophosphamide and total body brain-sparing irradiation, 1400 cGY. Graft-versus host disease prophylaxis by elutriation (n=9), or with cyclosporine + methotrexate or methylprednisolone (n=5). | Median visual acuity loss post HCT 0.36 log/MAR units (range 0-1.90).  Factors that correlated with loss of visual acuity after HCT: pretransplant MRI severity score >11 (p=0.03), pretransplant performance IQ score <76 (p=0.02), and the presence of pretransplant parieto-occipital demyelination on MRI (p=0.03). Patients with any one of these pretransplant characteristics had a median visual acuity loss of 1.00 log/MAR units.  NB data for additional predictors of visual acuity loss available. |
| Loes 1994 [42] | Diagnosis based on positive biochemical testing, abnormal neuropsychologic testing, and positive brain MRI. | Bone marrow transplantation. | Of the 4 patients followed at least 2 years before transplantation, 3 showed progressive disease, 1 showed stabilization.  Mean rate of MR severity (score change per month) in no-transplant group (n=21) = 0.24 with SD of 0.27. Mean rate of MR severity of the successful transplant group 0.059 with SD of 0.32. |
| Mahmood 2007 [19] | Neurological disability assessed with 4-point neurological deficit score (Peters et al 2004) – vision, hearing, speech, gait, fine motor skills, activities of daily living. MRI abnormality assessed using Loes score. Neurological deficit score 0 to 1 (single deficit) and MRI severity score <9 defined as mildly involved; 2 or more neurological deficits (score of 2) with MRI severity score of 9 or more defined as severely involved. | See Peters et al 2004 for details of HCT subset. | Untreated (n=30) versus treated (n=19) groups. Death: 13 (43%) versus 1 (5%). Age at death: 12.3 (4.9) versus 6.1 years. MRI severity at baseline: 4 (2.4) versus 3.5 (2.6). 5 year survival probability from date of baseline MRI: 54% versus 94.7. 10 year survival probability from date of baseline MRI: 42% (n=5) versus 94.7% (n=2). Number of patients followed for <10 years: 5 versus 6. Age in years of patients alive at last follow-up mean (SD): 13.2 (7.0) versus 15 (3.3). Duration in years of follow-up of survivors, mean (SD): 6.3 (6.1) versus 7.9 (4.0). NB t tests, p values and Chi square values available.  Survival probability for entire cohort (n=283): 66%. Moderate correlation between age of onset and 5 year survival. No significant difference in survival of the 92 (35%) of patients who had received Lorenzo’s Oil at some time during course of illness compared with 172 who had not. Adrenal function status known for184 patients. 167 (91%) had adrenal insufficiency. Probability of adrenal insufficiency not different in the group with the fatal outcome. Functional status deteriorated rapidly in most patients. 79/84 (94%) patients had a neurological deficit score of 2 or more at 5 years after onset of symptoms. 5/84 (6%) maintained a neurological deficit score of 0 or 1 in the 5 years after onset. Of 127 boys with greater than 5 years follow-up from onset of cerebral disease, 18 (14.2%) remained completely stable both neurologically and behaviourally during mean follow-up of 12.7 years. |
| Martin 2006 [36] | Diagnosis confirmed by lack of specific enzyme activity in peripheral blood leukocytes or fibroblasts. | Un-matched cord blood transplantation.  Conditioning regimen: oral busulfan, cyclophosphamide, antithymocyte globulin. Prophylaxis against GVHD cyclosporine and methylprednisolone. | Outcomes not reported separately for ALD patients. |
| Miller 2011 [40] | A diagnosis of ALD based on abnormal plasma VLCFA profile findings and the presence of active cerebral disease evidenced by characteristic white matter signal changes on brain MRI. Verbal Intelligence Quotient, Performance IQ, Full Scale IQ. Normalised measures of neuropsychometric assessment generated from age and clinically appropriate assessment tools e.g. Wechsler Intelligence Scale, Wechsler Adult Intelligence Scale. Radiographic severity of cerebral involvement determined by Loes score. Location of cerebral disease 8/60 (13%) predominant frontal, 49/60 (82%) predominant parieto-occipital, 3/60 (5%) mixed. | Allogenic hematopoietic cell transplantation. Donor type: related marrow 18/60 (30%), unrelated marrow 10/60 (17%), unrelated UCB (single) 12/60 (20%), unrelated UCB (double 20/60 (33%). HLA compatibility: matched 27/60 (45%), mismatched 33/60 (55%).  Conditioning regimen: varied by institution. Early patients underwent cyclophosphamide body irradiation, busulfan. From 2006, boys with advanced disease treated with reduced-intensity conditioning, boys with minimal cerebral disease continued with full body preparative regimen. | 47/60 (78%) patients alive at median follow-up (3.7 years). Causes of death disease progression n=5 (38% of deaths), graft failure n=3 (23%), infection n=2 (15%), hemorrage, aGVHD, haemolytic anemia n =1 for each (8%). Estimated probability of survival 75% (95% CI, 64-88%). Cumulative incidence of transplantation-relation mortality by day 100 8% (95% CI, 1%-15%).  Survival varied by Loes score at time of HCT. Probability of 5-year survival with baseline Loes score <10 was 89% (95% CI, 70%-96%), baseline Loes score >10 was 60% (95% CI, 34-78%). Probability of 5-year survival for baseline NFS=0 was 91% (95% CI, 69%-98%), baseline NFS >1 was 66% (95% CI 46-81%).  NB more data included e.g. neurologic outcomes and progression. |
| Peters 2004 [34] | Elevated concentrations of fasting plasma VLCFA. All but 2 had evidence of cerebral disease by magnetic resonance imaging of the brain. Neurologic examination, neuropsychological testing, and/or ALD-disability rating scale evaluation (ALD-DRS, 0-IV with increasing disability). Deficit in neurologic function in vision, hearing, speech, gait, and/or other areas eg fine motor skills and activities in daily living. 0 = no deficits, 1 = single deficit, 2 = 2 deficits, >2 = more than 2 deficits. Neuropsychological function by age appropriate Wechsler Intelligence Scale. Neuroradiologic assessment by Loes score. | Hematopoietic cell transplant. Donor type: Related donor n=42, of which 42/0 marrow/UCB, matched 33/0, mismatched 9/0. Unrelated donor n=52, of which 40/12 marrow/UCB, 27/4 matched, 13/8 mismatched.  Conditioning regimen: chemotherapy only n=48 (51%), chemotherapy and radiation n=46 (49%). | 59/94 patients alive at median 3.1 years (range 0.4 to 11.2 years). Estimated 5- to 8- year survival probabilities were both 56% (95% CI 44-68%). After RD HCT, 29/42 patients were alive at median 5.0 years (range 0.5-11.2 years). After URD HCT, 30 of 52 patients were alive at median 2.4 years (range 0.4-7.8 years). Estimated 5-year survival probabilities for RD and URD HCT were 64% (95% CI, 47-80%) and 53% (95% CI, 38-68%) respectively. Leading causes of death were progression of cerebral X-ALD (n=21), and GVHD (n=5). Overall 5 year survival varied with number of neurologic deficits before HCT: 0 deficits (n=32)= 70% (95 CI 51-89%), 1 neurologic deficit (n=28), 67% (95% CI 45-89%), 2 or more (n=30), 35% (95% CI, 14-57%). Overall 5- year survival varied by ALD DRS prior to HCT: level 0 93% (95% CI 79-100%), level 1 57% (95% CI, 32-81%), level 2 or greater 44% (95% CI 25-64%), and MRI severity score prior to HCT, score less than 9. (n=32), 84% (95% CI, 71-97%), score 9 or greater (n=34), 42% (95% CI, 18-66%). |
| Petryk 2012 [44] | Diagnosis based on presence of increased plasma VLCFA levels and characteristic white matter changes on MRI. | Hematopoietic cell transplantation. Study tested for adrenal insufficiency 1 year post HCT. Cord blood n=9, related marrow n=9, unrelated marrow n=2 | 16 patients manifested adrenal insufficiency before HCT. 0/16 showed any recovery after HCT. 3/4 patients that had normal adrenal function before HCT progressed to adrenal insufficiency after HCT. 1 patient had normal adrenal function before and after HCT. |
| Prasad 2008 [38] | Diagnoses confirmed by enzyme or substrate analysis in the peripheral blood or skin fibroblasts. DNA mutation analyses performed whenever possible. | Partially HLA-mismatched unrelated donor umbilical cord blood transplantation. | No individual results for ALD patients. |
| Sakata 2004 [35] | N/R | Unrelated donor marrow transplantation | 7 ALD patients engrafted, 6/7 survived. No time of follow-up given for ALD. |
| Shapiro 2000 [43] | Diagnoses by typical abnormalities of cerebral X-ALD on MRI, an IQ score over 70 on a standard measure, and moderate but not rapid deterioration on MRI scan and neuropsychological testing. Baseline and follow-up assessments by MRI (Loes scores), neurologic examination, neuropsychological tests (age appropriate Wechsler IQ Scale, language, nonverbal ability, auditory processing, visual processing, attention, memory, visual motor, motor speed, reaction time), visual evoked responses, and determinations of plasma concentrations of VLCFAs. | Bone marrow transplantation. HLA matched unrelated donors n=2, fully HLA-identical sibling donor n=10. | Corticospinal signs or mild dystonia resolved in 3/5 patients, unchanged in 2/5. Individual level improvements reported for neurologic and neuropsychological outcomes. 12/12 patients continue to have adrenal insufficiency posttransplant. |
| Wilken 2003 [45] | ‘Biochemical diagnosis’ | 10/12 bone marrow transplantation, 2/12 peripheral blood stem cells.  Preparative regimen in all patients using busulfan and cyclophosphamide in combination with antithymocyte and antilymphocyte globin in 10/12 patients. | 5/12 patients ‘good clinical outcome’. Follow-up MRI identified mild white matter changes, none of these patients developed neurologic or neuropsychologic disturbances for at least one year after HSCT. No signs of disease progression towards cALD. All boys in this group were diagnosed during early asymptomatic phases.  5/12 patients ‘poor clinical outcome’, characterised by initial deterioration of neurologic or neuropsychologic functions followed by stabilisation at a lower level about one year after transplantation. This grou had moderate manifestations before HSCT. Clinical disabilities after HSCT = loss of vision, inability to walk without support.  2/12 patients died due to disease progression at 6 and 28 months after BMT. Severe neurologic and neuropsychologic symptoms before HSCT. |

Appendix 1. Table of excluded studies.

|  | Paper | Reason for exclusion from further consideration |
| --- | --- | --- |
| 1 | Orchard 2010 | Discussion paper |
| 2 | Dunne 1993 | Case study |
| 3 | Cartier 2010 | Conference abstract |
| 4 | Shapiro 1997 | Conference abstract |
| 5 | Auluck2007 | Conference abstract |
| 6 | Moser 2003 | Lorenzo’s oil |
| 7 | Moser 2005 | Lorenzo’s oil |
| 8 | Matsukawa 2010 | Case study |
| 9 | Fatemi 2003 | MRI outcomes not required for model |
| 10 | Loes 2003 | MRI outcomes not required for model |
| 11 | Raymond 2007 | No new data, see Hubbard |
| 12 | van Geel 2014 | Single symptom (myelopathy) |
| 13 | Moser 2004 | Review |
| 14 | Lecumberri 2012 | Case study |
| 15 | Khan 2010 | Case study |
| 16 | Eichler 2007 | MRI outcomes not required for model |
| 17 | Costakos 1991 | Attitudes towards screening only |
| 18 | Matern 2013 | Review |
| 19 | Matern 2015 | Review and no new data for ALD |
| 20 | Melham 2000 | MRI outcomes not required for model |
| 21 | Polgreen 2011 | Outcomes by early diagnosis, importance of diagnosis of unexplained adrenal insufficiency, exclude |
| 22 | Shimozawa 2011 | Mutation analysis, no useful screening data |
| 23 | Silveri 2004 | Not prenatal or newborn screening |
| 24 | Boehm 1999 | Diagnostic test of carrier status by DNA analysis |
| 25 | Gigarel 2004 | N=1 ALD |
| 26 | Tolar 2007 | N=3 |
| 27 | Singh 1998 | Lovastin therapy |
| 28 | Shih 2013 | Statin treatment |
| 29 | Salsano 2012 | Case study |
| 30 | Renaud 2009 | Overview of programme for patients with X-ALD/AMN only. No data. |
| 31 | Rockenbach 2012 | Biomarker outcomes only. |
| 32 | Moser 2005 | Lorenzo’s Oil |
| 33 | Pai 2004 | Lovastatin therapy |
| 34 | McKinney 2013 | DTI parameters versus Loes score, neurologic function scores and neuropsychological scores, not useful data for model |
| 35 | Fitzpatrick 2008 | Case study |
| 36 | Cartier 2019 | No data |
| 37 | Boelens 2011 | Comment piece |
| 38 | Awaya 2011 | Case study |

Appendix 2. Quality Assessment – CASP checklist for cohort studies, summary of assessments for studies supplying data for the model.

| Mahmood 2007 [19] | Yes | Can’t tell | No |
| --- | --- | --- | --- |
| Did the study address a clearly focused question? |  |  |  |
| Was the cohort recruited in an acceptable way? |  |  |  |
| Was the exposure accurately measured to minimise bias? |  |  |  |
| Was the outcome accurately measured to minimise bias? |  |  |  |
| Have the authors identified all important confounding factors? |  |  |  |
| Have the authors taken account of the important confounding factors in the design? |  |  |  |
| Was the follow up of subjects complete enough? |  |  |  |
| Was the follow up of subjects long enough? |  |  |  |
| What are the results? | 46% of non-transplanted patients died during the mean follow-up period of 5.9 years. | | |
| How precise are the results? | Precise | | |
| Do you believe the results? |  |  |  |
| Can the results be applied to the local population? |  |  |  |
| Do the results of this study fit with other available evidence? |  |  |  |
| What are the implications of this study for practice? | Early transplantation is beneficial | | |

| Peters 2004 [34] | Yes | Can’t tell | No |
| --- | --- | --- | --- |
| Did the study address a clearly focused question? |  |  |  |
| Was the cohort recruited in an acceptable way? |  |  |  |
| Was the exposure accurately measured to minimise bias? |  |  |  |
| Was the outcome accurately measured to minimise bias? |  |  |  |
| Have the authors identified all important confounding factors? |  |  |  |
| Have the authors taken account of the important confounding factors in the design? |  |  |  |
| Was the follow up of subjects complete enough? |  |  |  |
| Was the follow up of subjects long enough? |  |  |  |
| What are the results? | The estimated 5-8 years survival was 56% | | |
| How precise are the results? | Good | | |
| Do you believe the results? |  |  |  |
| Can the results be applied to the local population? |  |  |  |
| Do the results of this study fit with other available evidence? |  |  |  |
| What are the implications of this study for practice? | Early transplantation is beneficial | | |

| Miller 2011 [40] | Yes | Can’t tell | No |
| --- | --- | --- | --- |
| Did the study address a clearly focused question? |  |  |  |
| Was the cohort recruited in an acceptable way? |  |  |  |
| Was the exposure accurately measured to minimise bias? |  |  |  |
| Was the outcome accurately measured to minimise bias? |  |  |  |
| Have the authors identified all important confounding factors? |  |  |  |
| Have the authors taken account of the important confounding factors in the design? |  |  |  |
| Was the follow up of subjects complete enough? |  |  |  |
| Was the follow up of subjects long enough? |  |  |  |
| What are the results? | 78% of transplanted patients were alive at 3.7 years follow-up. | | |
| How precise are the results? | Precise | | |
| Do you believe the results? |  |  |  |
| Can the results be applied to the local population? |  |  |  |
| Do the results of this study fit with other available evidence? |  |  |  |
| What are the implications of this study for practice? | Early transplantation (before cerebral involvement) is beneficial | | |

| De Beer 2014 [25] | Yes | Can’t tell | No |
| --- | --- | --- | --- |
| Did the study address a clearly focused question? |  |  |  |
| Was the cohort recruited in an acceptable way? |  |  |  |
| Was the exposure accurately measured to minimise bias? |  |  |  |
| Was the outcome accurately measured to minimise bias? |  |  |  |
| Have the authors identified all important confounding factors? |  |  |  |
| Have the authors taken account of the important confounding factors in the design? |  |  |  |
| Was the follow up of subjects complete enough? |  |  |  |
| Was the follow up of subjects long enough? |  |  |  |
| What are the results? | 63% of patients with AMN developed cerebral demyelination 10.2 years after onset of myelonopathy | | |
| How precise are the results? | Possible ascertainment bias | | |
| Do you believe the results? |  |  |  |
| Can the results be applied to the local population? |  |  |  |
| Do the results of this study fit with other available evidence? |  |  |  |
| What are the implications of this study for practice? | Demyelination in AMN is more frequent than previously considered. More therapies are needed to treat this. | | |

| Keller 2012 [26] | Yes | Can’t tell | No |
| --- | --- | --- | --- |
| Did the study address a clearly focused question? |  |  |  |
| Was the cohort recruited in an acceptable way? |  |  |  |
| Was the exposure accurately measured to minimise bias? |  |  |  |
| Was the outcome accurately measured to minimise bias? |  |  |  |
| Have the authors identified all important confounding factors? |  |  |  |
| Have the authors taken account of the important confounding factors in the design? |  |  |  |
| Was the follow up of subjects complete enough? | N/A | N/A | N/A |
| Was the follow up of subjects long enough? | N/A | N/A | N/A |
| What are the results? | Severity of AMN can be evaluated using a staging method based on weakness. | | |
| How precise are the results? | Precise | | |
| Do you believe the results? |  |  |  |
| Can the results be applied to the local population? |  |  |  |
| Do the results of this study fit with other available evidence? |  |  |  |
| What are the implications of this study for practice? | Staging can be used to develop rehabilitative techniques. | | |

| Engelen 2014 [29] | Yes | Can’t tell | No |
| --- | --- | --- | --- |
| Did the study address a clearly focused question? |  |  |  |
| Was the cohort recruited in an acceptable way? |  |  |  |
| Was the exposure accurately measured to minimise bias? |  |  |  |
| Was the outcome accurately measured to minimise bias? |  |  |  |
| Have the authors identified all important confounding factors? |  |  |  |
| Have the authors taken account of the important confounding factors in the design? |  |  |  |
| Was the follow up of subjects complete enough? |  |  |  |
| Was the follow up of subjects long enough? |  |  |  |
| What are the results? | Female carriers of X-ALD develop signs and symptoms of myelopathy and peripheral neuropathy. Frequency of symptoms increases with age. | | |
| How precise are the results? | Possible bias towards symptomatic women | | |
| Do you believe the results? |  |  |  |
| Can the results be applied to the local population? |  |  |  |
| Do the results of this study fit with other available evidence? |  |  |  |
| What are the implications of this study for practice? | Mutation analysis of ABCD1 should be considered for non-compressive myelopathy. | | |

Reference List

1. Kaltenthaler E, Tappenden P, Paisley S, Squires H. NICE DSU Technical Support Document 13: Identifying and reviewing evidence to inform the conceptualisation and population of cost-effectiveness models. 2011. <http://www.nicedsu.org.uk>. Accessed 14^th^ October 2014
2. Bezman L, Moser AB, Raymond GV, Rinaldo P, Watkins PA, Smith KD et al. Adrenoleukodystrophy: incidence, new mutation rate, and results of extended family screening**.** Annals of Neurology 2001, 49**:** 512-517.
3. Di Biase A, Salvati S, Avellino C, Cappa M, Bertini E, Moroni I et al. X-linked adrenoleukodystrophy: first report of the Italian Study Group**.** Italian Journal of Neurological Sciences 1998;19**:**315-319.
4. Heim P, Claussen M, Hoffmann B, Conzelmann E, Gartner J, Harzer K et al. Leukodystrophy incidence in Germany**.** American Journal of Medical Genetics 1997;71**:**475-478.
5. Horn MA, Retterstol L, Abdelnoor M, Skjeldal OH, Tallaksen CM. Adrenoleukodystrophy in Norway: high rate of de novo mutations and age-dependent penetrance**.** Pediatric Neurology 2013;48**:**212-219.
6. Jardim LB, da Silva AC, Blank D, Villanueva MM, Renck L, Costa ML et al. X-linked adrenoleukodystrophy: clinical course and minimal incidence in South Brazil**.** Brain & Development 2010;32**:**180-190.
7. Kirk EP, Fletcher JM, Sharp P, Carey B, Poulos A. X-linked adrenoleukodystrophy: the Australasian experience**.** American Journal of Medical Genetics 1998;76**:**420-423.
8. Sereni C, Paturneau-Jouas M, Aubourg P, Baumann N, Feingold J. Adrenoleukodystrophy in France: an epidemiological study**.** Neuroepidemiology 1993;12**:**229-233.
9. Takemoto Y, Suzuki Y, Tamakoshi A, Onodera O, Tsuji S, Hashimoto T et al. Epidemiology of X-linked adrenoleukodystrophy in Japan**.** Journal of Human Genetics 2002;47**:**590-593.
10. Van Geel BM, Assies J, Weverling GJ, Barth PG. Predominance of the adrenomyeloneuropathy phenotype of X-linked adrenoleukodystrophy in The Netherlands: a survey of 30 kindreds**.** Neurology 1994;44**:**2343-2346.
11. Pereira FS, Matte U, Habekost CT, de Castilhos RM, El Husny AS, Lourenco CM et al. Mutations, clinical findings and survival estimates in South American patients with X-linked adrenoleukodystrophy**.** PLoS ONE 2012;7**:**e34195.
12. Ruiz M, Coll MJ, Pampols T, Giros M. X-linked adrenoleukodystrophy: phenotype distribution and expression of ALDP in Spanish kindreds**.** American Journal of Medical Genetics 1998;76**:**424-427.
13. Moser HW. Adrenoleukodystrophy: phenotype, genetics, pathogenesis and therapy**.** Brain 1997;120**:**1485-1508.
14. Lan F, Wang Z, Ke L, Xie H, Huang L, Huang H et al. A rapid and sensitive protocol for prenatal molecular diagnosis of X-linked adrenoleukodystrophy**.** Clinica Chimica Acta 2010;411**:**1992-1997.

15. Hubbard WC, Moser AB, Tortorelli S, Liu A, Jones D, Moser H. Combined liquid chromatography-tandem mass spectrometry as an analytical method for high throughput screening for X-linked adrenoleukodystrophy and other peroxisomal disorders: preliminary findings**.** Molecular Genetics & Metabolism 2006;89**:**185-187.

16. Hubbard WC, Moser AB, Liu AC, Jones RO, Steinberg SJ, Lorey F et al. Newborn screening for X-linked adrenoleukodystrophy (X-ALD): validation of a combined liquid chromatography-tandem mass spectrometric (LC-MS/MS) method**.** Molecular Genetics & Metabolism 2009;97**:**212-220.

17. Theda C, Gibbons K, DeFor TE, Donohue PK, Golden WC, Kline AD et al. Newborn screening for X-linked adrenoleukodystrophy: Further evidence high throughput screening is feasible**.** Molecular Genetics and Metabolism 2014;111**:**55-57.

18. Turgeon CT, Moser AB, Mørkrid L, Magera MJ, Gavrilov DK, Oglesbee D et al. Streamlined determination of lysophosphatidylcholines in dried blood spots for newborn screening of X-linked adrenoleukodystrophy**.** Molecular Genetics and Metabolism 2015;114**:**46-50.

19. Mahmood A, Raymond GV, Dubey P, Peters C, Moser HW. Survival analysis of haematopoietic cell transplantation for childhood cerebral X-linked adrenoleukodystrophy: a comparison study**.** Lancet Neurology 2007;6**:**687-692.

20. Suzuki Y, Takemoto Y, Shimozawa N, Imanaka T, Kato S, Furuya H et al. Natural history of X-linked adrenoleukodystrophy in Japan**.** Brain & Development 2005;27**:**353-357.

21. Liang JS, Lee WT, Hwu WL, Peng SS, Chu LW, Wang PJ et al. Adrenoleukodystrophy: clinical analysis of 9 Taiwanese children**.** Acta Paediatr Taiwan 2004;45**:**272-277.

22. Moser HW, Bezman L, Lu SE, Raymond GV. Therapy of X-linked adrenoleukodystrophy: prognosis based upon age and MRI abnormality and plans for placebo-controlled trials. Journal of Inherited Metabolic Disease 2000;23**:**273-277.

23. Stephenson DJ, Bezman L, Raymond GV. Acute presentation of childhood adrenoleukodystrophy**.** Neuropediatrics 2000;31**:**293-297.

24. Winstone A, Stellitano L, Verity C. The presentation and diagnosis of UK children with X-lined adrenoluekodystrophy ascertained over 17 years**.** Developmental Medicine & Child Neurology 2015;57**:**2.

25. De Beer M, Engelen M, Van Geel BM. Frequent occurrence of cerebral demyelination in adrenomyeloneuropathy**.** Neurology 2014;83**:**2227-2231.

26. Keller JL, Wang JI, Kang JY, Hanson JA, Kamath P, Swain JO et al. Strength: a relevant link to functional performance in the neurodegenerative disease of adrenomyeloneuropathy**.** Neurorehabilitation & Neural Repair 2012;26**:**1080-1088.

27. Walterfang MA, O'Donovan J, Fahey MC, Velakoulis D. The neuropsychiatry of adrenomyeloneuropathy**.** Cns Spectrums 200;12**:**696-701.

28. Zachowski K, Dubey P, Raymond G, Mori S, Bastian A. Sensorimotor function and axonal integrity in adrenalmyeloneuropathy. Archives of Neurology 2006;63:74-80.

29. Engelen M, Barbier M, Dijkstra IME, Schur R, De Bie RMA, Verhamme C et al. X-linked adrenoleukodystrophy in women: A cross-sectional cohort study**.** Brain 2014;137**:**693-706.

30. Kaga M, Furushima W, Inagaki M, Nakamura M. Early neuropsychological signs of childhood adrenoleukodystrophy (ALD)**.** Brain & Development 2009;31**:**558-561.

31. Dubey P, Raymond GV, Moser AB, Kharkar S, Bezman L, Moser HW. Adrenal insufficiency in asymptomatic adrenoleukodystrophy patients identified by very long-chain fatty acid screening**.** Journal of Pediatrics 2005;146**:**528-532.

32. Cox CS, Dubey P, Raymond GV, Mahmood A, Moser AB, Moser HW. Cognitive evaluation of neurologically asymptomatic boys with X-linked adrenoleukodystrophy**.** Archives of Neurology 2006;63**:**69-73.

33. Gess A, Christiansen SP, Pond D, Peters C. Predictive factors for vision loss after hematopoietic cell transplant for X-linked adrenoleukodystrophy**.** Journal of Aapos: American Association for Pediatric Ophthalmology & Strabismus 2008;12**:**273-276.

34. Peters C, Charnas LR, Tan Y, Ziegler RS, Shapiro EG, DeFor T et al. Cerebral X-linked adrenoleukodystrophy: the international hematopoietic cell transplantation experience from 1982 to 1999. Blood 2004;104**:**881-888.

35. Sakata N, Kawa K, Kato K, Yabe H, Yabe M, Nagasawa M et al. Unrelated donor marrow transplantation for congenital immunodeficiency and metabolic disease: an update of the experience of the Japan Marrow Donor Program**.** International Journal of Hematology 2004;80**:**174-182.

36. Martin PL, Carter SL, Kernan NA, Sahdev I, Wall D, Pietryga D et al. Results of the cord blood transplantation study (COBLT): outcomes of unrelated donor umbilical cord blood transplantation in pediatric patients with lysosomal and peroxisomal storage diseases**.** Biology of Blood & Marrow Transplantation 2006;12**:**184-194.

37. Gassas A, Raiman J, White L, Schechter T, Clarke J, Doyle J. Long-term adaptive functioning outcomes of children with inherited metabolic and genetic diseases treated with hematopoietic stem cell transplantation in a single large pediatric center: parents' perspective**.** Journal of Pediatric Hematology/Oncology 2011;33**:**216-220.

38. Prasad V, Mendizabal A, arikh SH, Szabolcs P, Driscoll T, Page K *et al*. Unrelated donor umbilical cord blood transplantation for inherited metabolic disorders in 159 pediatric patients from a single center: influence of cellular composition of the graft on transplantation outcomes. Blood 2008;112:2979-2989.

39. Baumann M, Korenke GC, Weddige-Diedrichs A, Wilichowski E, Hunneman DH, Wilken B et al. Haematopoietic stem cell transplantation in 12 patients with cerebral X-linked adrenoleukodystrophy**.** European Journal of Pediatrics 2003;162**:**6-14.

40. Miller W, Rothman S, Nascene D, Kivisto T, DeFor T, Ziegler R et al. Outcomes after allogenic hematopoetic cell transplantation for childhood cerebral adrenoleukodystrophy: the largest single-institution cohort report**.** Blood 2011;118**:**1971-1978.

41. Beam D, Poe MD, Provenzale JM, Szabolcs P, Martin PL, Prasad V et al. Outcomes of unrelated umbilical cord blood transplantation for X-linked adrenoleukodystrophy**.** Biology of Blood & Marrow Transplantation 2007;13**:**665-674.

42. Loes DJ, Stillman AE, Hite S, Shapiro E, Lockman L, Latchaw RE et al. Childhood cerebral form of adrenoleukodystrophy: short-term effect of bone marrow transplantation on brain MR observations**.** Ajnr: American Journal of Neuroradiology 1994;15**:**1767-1771.

43. Shapiro E, Krivit W, Lockman L, Jambaque I, Peters C, Cowan M et al. Long-term effect of bone-marrow transplantation for childhood-onset cerebral X-linked adrenoleukodystrophy**.** Lancet 2000;356**:**713-718.

44. Petryk A, Polgreen LE, Chahla S, Miller W, Orchard PJ. No evidence for the reversal of adrenal failure after hematopoietic cell transplantation in X-linked adrenoleukodystrophy**.** Bone Marrow Transplantation 2012;47**:**1377-1378.

45. Wilken B, Dechent P, Brockmann K, Finsterbusch J, Baumann M, Ebell W et al. Quantitative proton magnetic resonance spectroscopy of children with adrenoleukodystrophy before and after hematopoietic stem cell transplantation**.** Neuropediatrics 2003;34**:**237-246.

46. Stellitano L, Winstone A, Van der Knaap M, Verity C. Leukodystrophies and genetic leukoencephalopathies in childhood: a national epidemiological study. Developmental Medicine & Child Neurology 2016;58:680-689
