# Supplementary material for: Economic impact of screening for X-linked Adrenoleukodystrophy within a newborn blood spot screening programme
Source: Orphanet J Rare Dis. 2018 Oct 11;13:179. doi: 10.1186/s13023-018-0921-4 (PMC6182830; doi:10.1186/s13023-018-0921-4)
Supplement: Supplementary file 3 — Calculation of the Quality Adjusted Life Years (QALYs). This file presents more detail on the calculations that were used to calculate the QALYs in the model presented in this manuscript. (DOCX 27 kb) [file 13023_2018_921_MOESM3_ESM.docx]

**Additional File 3: Calculation of the Quality Adjusted Life Years (QALYs)**

To estimate the quality adjusted life year (QALYs) for patients with CCALD the ALD-DRS was mapped onto the EQ-5D-5L as shown in Table 1 [1,2]. A proportional decrement for each ALD-DRS state was calculated by the difference in the overall QALY for the ALD-DRS health state and the constant. The proportional decrement was then applied to the age specific general population means [3] to give age specific QALYs (as shown in Table 2). A similar approach is taken for patients with AMN and women with X-ALD. QALY values were taken from a study of MS [4] for EDSS states 3 and 6 and the difference between them and the general population mean for the age bracket of 45-55, the mean age in the study, were calculated as shown in Table 2. No uncertainty was included for the proportional decrements. Instead uncertainty was incorporated for the baseline EDSS state value as shown in Table 3 for AMN and women with X-ALD. For CCALD patients uncertainty was included for each level of each dimension of the EQ-5D-5L based on the publish confidence intervals and using a normal distribution as shown in Table 4 [2].

Table 1: Mapping of the ALD-DRS onto the EQ-5D-5L

|  | **Decrement applied to the constant** | **ALD-DRS I** | **ALD-DRS II** | **ALD-DRS III** | **ALD-DRS IV** |
| --- | --- | --- | --- | --- | --- |
| Constant | 1.003 |  |  |  |  |
| Mobility = 2 | 0.057 | 0.946 |  |  |  |
| Mobility = 3 | 0.075 |  | 0.928 |  |  |
| Mobility = 4 | 0.208 |  |  | 0.795 |  |
| Mobility = 5 | 0.255 |  |  |  | 0.748 |
| Self-care = 2 | 0.058 | 0.888 |  |  |  |
| Self-care = 3 | 0.083 |  | 0.845 |  |  |
| Self-care = 4 | 0.176 |  |  | 0.619 |  |
| Self-care = 5 | 0.208 |  |  |  | 0.54 |
| Usual activities = 2 | 0.048 | 0.84 |  |  |  |
| Usual activities = 3 | 0.067 |  | 0.778 |  |  |
| Usual activities = 4 | 0.165 |  |  | 0.454 |  |
| Usual activities = 5 | 0.165 |  |  |  | 0.375 |
| Pain/discomfort = 2 | 0.059 | 0.781 |  |  |  |
| Pain/discomfort = 3 | 0.08 |  | 0.698 |  |  |
| Pain/discomfort = 4 | 0.245 |  |  | 0.209 | 0.13 |
| Pain/discomfort = 5 | 0.298 |  |  |  |  |
| Anxiety/depression = 2 | 0.073 |  |  |  |  |
| Anxiety/depression = 3 | 0.099 | 0.682 | 0.599 | 0.11 | 0.031 |
| Anxiety/depression = 4 | 0.282 |  |  |  |  |
| Anxiety/depression = 5 | 0.282 |  |  |  |  |
| **Overall QALY** |  | **0.682** | **0.599** | **0.11** | **0.031** |

Table 2: Proportional decrements applied in the model

|  |  | **ALD-DRS1** | **ALD-DRS2** | **ALD-DRS3** | **ALD-DRS4** | **EDSS 3** | **EDSS 6** |
| --- | --- | --- | --- | --- | --- | --- | --- |
|  | **Proportional**  **Decrement**  **General**  **population means** | 0.320 | 0.403 | 0.890 | 0.969 | 0.179 | 0.390 |
| **Under 25** | 0.94 | 0.639 | 0.561 | 0.103 | 0.029 | 0.772 | 0.574 |
| **25-34** | 0.93 | 0.632 | 0.555 | 0.102 | 0.029 | 0.764 | 0.568 |
| **35-44** | 0.91 | 0.619 | 0.544 | 0.100 | 0.028 | 0.747 | 0.556 |
| **45-55** | 0.85 | 0.578 | 0.508 | 0.093 | 0.026 | 0.698 | 0.519 |
| **55-64** | 0.8 | 0.544 | 0.478 | 0.088 | 0.025 | 0.657 | 0.488 |
| **65-74** | 0.78 | 0.530 | 0.466 | 0.086 | 0.024 | 0.641 | 0.476 |
| **75+** | 0.73 | 0.496 | 0.436 | 0.080 | 0.023 | 0.600 | 0.390 |

Table 3: Uncertainty in the EDSS state values

| **Utilities** | **Mean** | **95% upper limit** | **95% lower limit** | **SE** | **Alpha** | **Beta** | **Gamma Distribution** |
| --- | --- | --- | --- | --- | --- | --- | --- |
| EDSS 3 | 0.70 | 0.80 | 0.61 | 0.048 | 210.37 | 0.003 | (210, 0.003) |
| EDSS 6 | 0.52 | 0.60 | 0.42 | 0.046 | 128.76 | 0.004 | (129, 0.004) |

Table 4: Uncertainty in the CCALD values

|  | **Mean** | **95% upper limit** | **95% lower limit** | **SE** | **Normal Distribution** |
| --- | --- | --- | --- | --- | --- |
| Constant | 1.003 | 1.019 | 0.983 | 0.0092 | (1.003,0.0092) |
| Mobility = 2 | 0.057 | 0.075 | 0.043 | 0.0082 | (0.057,0.0082) |
| Mobility = 3 | 0.075 | 0.093 | 0.057 | 0.0092 | (0.075,0.0092) |
| Mobility = 4 | 0.208 | 0.227 | 0.19 | 0.0094 | (0.208,0.0094) |
| Mobility = 5 | 0.255 | 0.275 | 0.237 | 0.0097 | (0.255,0.0097) |
| Self-care = 2 | 0.058 | 0.074 | 0.045 | 0.0074 | (0.058,0.0074) |
| Self-care = 3 | 0.083 | 0.101 | 0.061 | 0.0102 | (0.083,0.0102) |
| Self-care = 4 | 0.176 | 0.197 | 0.157 | 0.0102 | (0.176,0.0102) |
| Self-care = 5 | 0.208 | 0.225 | 0.189 | 0.0092 | (0.208,0.0092) |
| Usual activities = 2 | 0.048 | 0.066 | 0.033 | 0.0084 | (0.048,0.0084) |
| Usual activities = 3 | 0.067 | 0.086 | 0.047 | 0.0099 | (0.067,0.0099) |
| Usual activities = 4 | 0.165 | 0.18 | 0.147 | 0.0084 | (0.165,0.0084) |
| Usual activities = 5 | 0.165 | 0.184 | 0.152 | 0.0082 | (0.165,0.0082) |
| Pain/discomfort = 2 | 0.059 | 0.075 | 0.042 | 0.0084 | (0.059,0.0084) |
| Pain/discomfort = 3 | 0.08 | 0.098 | 0.059 | 0.0099 | (0.08,0.0099) |
| Pain/discomfort = 4 | 0.245 | 0.264 | 0.225 | 0.0099 | (0.245,0.0099) |
| Pain/discomfort = 5 | 0.298 | 0.317 | 0.278 | 0.0099 | (0.298,0.0099) |
| Anxiety/depression = 2 | 0.073 | 0.089 | 0.058 | 0.0079 | (0.073,0.0079) |
| Anxiety/depression = 3 | 0.099 | 0.119 | 0.079 | 0.0102 | (0.099,0.0102) |
| Anxiety/depression = 4 | 0.282 | 0.298 | 0.263 | 0.0089 | (0.282,0.0089) |
| Anxiety/depression = 5 | 0.282 | 0.3 | 0.267 | 0.0084 | (0.282,0.0084) |

Reference List

1. Peters C, Charnas LR, Tan Y, Ziegler RS, Shapiro EG, DeFor T et al. Cerebral X-linked adrenoleukodystrophy: the international hematopoietic cell transplantation experience from 1982 to 1999. Blood 2004;104**:**881-888.

2. Devlin N, Van Hout B. An EQ-5D-5L value set for England. Office of Health Economics. https://www.ohe.org/publications/valuing-health-related-quality-life-eq-5d-5l-value-set-england. Accessed on 23^rd^ March 2015

3. Kind P, Dolan P, Gudex C, Williams A. Variations in population health status: results from a United Kingdom national questionnaire survey**.** BMJ 1998;316**:**736.

4. Kobelt G, Berg J, Lindgren P, Fredrikson S, Jönsson B. Costs and quality of life of patients with multiple sclerosis in Europe**.** J Neurol Neurosurg Psychiatry 2006;77**:**918.
