# Supplementary material for: Economic impact of screening for X-linked Adrenoleukodystrophy within a newborn blood spot screening programme
Source: Orphanet J Rare Dis. 2018 Oct 11;13:179. doi: 10.1186/s13023-018-0921-4 (PMC6182830; doi:10.1186/s13023-018-0921-4)
Supplement: Supplementary file 4 — Calculation of the costs and their sources. This file contains a table that outlines the included costs for the model presented in this manuscript and their sources. (DOCX 48 kb) [file 13023_2018_921_MOESM4_ESM.docx]

**Additional File 4: Calculation of the costs and their sources**

| **X-ALD Phenotype** | **Parameter** | **Parameter components** | **Cost (£) 2014/15 prices** | | **Reference** |
| --- | --- | --- | --- | --- | --- |
|  |  |  | **Child** | **Adult** |  |
| Screening | Screening test | Marginal cost of the additional test for X-ALD per baby | **£0.5** |  | [1] |
|  | Screen positive cost |  | **£500** |  | [1] |
| Non X-ALD Screen positive cases | Non – X-ALD Screen positive | Plasma VLFA levels test | £85 |  | [2] |
|  | Diagnosis and Monitoring costs | ABCD1 mutation analysis | £450 |  | [3] |
|  |  | PEX mutation analysis | £600 |  | [3] |
|  |  | Metabolic specialist | £403 |  | [4] |
|  |  | Paediatric neurology x4 | £1,209 |  | [4] |
|  |  | Occupational therapy x 4 | £256 |  | [4] |
|  |  | Gastroenterology x 4 | £802 |  | [4] |
|  |  | Speech x4 | £335 |  | [4] |
|  |  | Hearing x 4 | £349 |  | [4] |
|  |  | **Total** | **£4,490** |  |  |
| Screen positive or asymptomatic | Diagnosis | Plasma VLFA levels test | £85 |  | [2] |
|  |  | ABCD1 mutation analysis | £450 |  | [3] |
|  |  | Metabolic specialist | £403 |  | [4] |
|  |  | Neurologist | £302 |  | [4] |
|  |  | Neuropsychology assessment | £379 |  | [4] |
|  |  | Eye sight and hearing tests | £177 |  | [4] |
|  |  | Nerve conduction tests | £391 |  | [4] |
|  |  | MRI scan | £149 |  | [4] |
|  |  | Endocrinologist | £242 |  | [4] |
|  |  | Genetic counselling for family to include siblings, parents (plus parents’ siblings and their children and grandparents) tests for 6 family members + 3 appointments with metabolic specialist | £2,155 |  | [3,4] |
|  |  | **Total** | **£4,735** |  |  |
|  | Monitoring | Annual endocrinologist | £242 | £144 | [4] |
|  |  | Annual neurology evaluation | £302 | £174 | [4] |
|  |  | Annual metabolic evaluation | £403 | £174 | [4] |
|  |  | Neuropsychology assessment | £379 | £379 | [4] |
|  |  | Eye sight and hearing tests | £177 | £165 | [4] |
|  |  | Nerve conduction tests | £391 | £139 | [4] |
|  |  | **Total (annual)** | **£1,895** | **£1,175** |  |
|  | From 6 month until 30 months | Annual brain MRI without contrast | £141 |  | [4] |
|  |  | 6 monthly serum ACTH and cortisol | £45 |  | [5] |
|  |  | **Total (annual)** | **£186** |  |  |
|  | From 36 months to 10 years | 6 monthly brain MRI with contrast | £406 |  | [4] |
|  |  | 6 monthly serum ACTH and cortisol | £45 |  | [5] |
|  |  | **Total (annual)** | **£451** |  |  |
|  | From 10 years to 18 | Annual brain MRI without contrast | £149 |  | [4] |
|  |  | 6 monthly serum ACTH and cortisol | £45 |  | [5] |
|  |  | **Total (annual)** | **£194** |  |  |
|  | 18+ | Annual serum ACTH and cortisol |  | £22 | [5] |
|  |  | Annual MRI without contrast |  | £143 | [4] |
|  |  | **Total (annual)** |  | **£165** |  |
| AMN | Pre Diagnosis | GP appointments x6 |  | £264 | [6] |
|  |  | Neurologist x 2 |  | £349 | [4] |
|  | Diagnosis | Plasma VLCFA levels test |  |  |  |
|  |  | ABCD1 mutation analysis |  | £450 | [3] |
|  |  | Metabolic specialist |  | £174 | [4] |
|  |  | Neurologist |  | £174 | [4] |
|  |  | Nerve conduction tests |  | £139 | [4] |
|  |  | MRI scan |  | £143 | [4] |
|  |  | Endocrinologist |  | £144 | [4] |
|  |  | Genetic counselling for 6 family members + 3 appointments with metabolic team |  | £2,155 | [3,4] |
|  |  | **Total** |  | **£4,077** |  |
|  | Monitoring | Annual serum ACTH and cortisol |  | £22 | [5] |
|  |  | Annual MRI without contrast |  | £143 | [4] |
|  |  | Urologist x1 per year |  | £99 | [4] |
|  |  | Physiotherapist x4 per year |  | £185 | [4] |
|  |  | Neurologist x1 per year |  | £174 | [4] |
|  |  | Metabolic specialist x1 per year |  | £174 | [4] |
|  |  | Nerve conduction tests |  | £139 | [4] |
|  |  | Orthotics |  | £109 | [4] |
|  |  | **Total (annual)** |  | **£1,046** |  |
|  | Care costs - Mild cases EDSS 2 | Services - includes home help/transportation |  | £88* | [7,8] |
|  |  | Investments - Aids, modifications etc |  | £138* | [7,8] |
|  |  | **Total (annual)** |  | **£226** |  |
|  | Care costs - Moderate/Severe EDSS 6 | Services - includes home help/transportation |  | £2,038* | [7,8] |
|  |  | Investments - Aids, modifications etc |  | £2,246* | [7,8] |
|  |  | **Total (annual)** |  | **£4,735** |  |
| Women | Pre Diagnosis | GP appointments x6 |  | £264 | [6] |
|  |  | Neurologist x 2 |  | £349 | [4] |
|  | Diagnosis | Plasma VLCFA levels test |  | £85 | [2] |
|  |  | ABCD1 mutation analysis |  | £450 | [3] |
|  |  | Metabolic specialist/neurologist |  | £174 | [4] |
|  |  | Nerve conduction tests |  | £139 | [4] |
|  |  | Genetic counselling for family to include children, siblings (and their children etc. if applicable) and parents - 6 tests for family members and 3 appointments with metabolic specialists |  | £2,155 | [3,4] |
|  |  | MRI scan |  | £143 | [4] |
|  |  | **Total** |  | **£3,759** |  |
|  | Monitoring | Consultant appointment yearly |  | £174 | [4] |
|  |  | Nerve conduction and blood test |  | £139 | [4] |
|  |  | **Total (annual)** |  | **£313** |  |
|  | Health costs over 40 years old | Consultant appointment yearly |  | £174 | [4] |
|  |  | Nerve conduction tests |  | £139 | [4] |
|  |  | Physiotherapist x4 per year |  | £185 | [4] |
|  |  | Urologist x 1 per year and treatment options |  | £99 | [4] |
|  |  | Metabolic specialist x 1 per year |  | £174 | [4] |
|  |  | Orthotics |  | £109 | [4] |
|  |  | **Total (annual)** |  | **£880** |  |
|  | Care costs - Mild cases EDSS 2 | Services - includes home help/transportation |  | £88* | [7,8] |
|  |  | Investments - Aids, modifications etc |  | £138* | [7,8] |
|  |  | **Total (annual)** |  | **£226** |  |
| Symptomatic X-ALD | Pre Diagnosis | GP appointments x6 | £264 |  | [6] |
|  |  | Neurologist x 2 | £604 |  | [4] |
|  | Diagnosis | Plasma VLCFA levels test | £85 |  | [2] |
|  |  | ABCD1 mutation analysis | £450 |  | [3] |
|  |  | Metabolic specialist/neurologist | £403 |  | [4] |
|  |  | Nerve conduction tests | £391 |  | [4] |
|  |  | MRI scan | £203 |  | [4] |
|  |  | Genetic counselling for family to include children, siblings (and their children etc. if applicable) and parents - 6 tests for family members and 3 appointments with metabolic specialists | £2,155 |  | [3,4] |
|  |  | Neuropsychology assessment | £379 |  | [4] |
|  |  | Endocrinologist | £242 |  | [4] |
|  |  | Eye sight and hearing tests | £177 |  | [4] |
|  |  | Nerve conduction tests | £391 |  | [4] |
|  |  | **Total** | **£5,923** |  |  |
| Mild/Moderate | Health care costs | Occupational therapist x 2 visits per year | £128 | £128 | [4] |
|  |  | Hearing test | £77 | £73 | [4] |
|  |  | Eyesight test | £100 | £92 | [4] |
|  |  | Nerve conduction studies | £391 | £139 | [4] |
|  |  | Neuropsychology assessment | £379 | £379 | [4] |
|  |  | Orthotics appointments x2 | £219 | £219 | [4] |
|  |  | Psychology x4 | £707 |  | [4] |
|  |  | Physiotherapist x12 visits per year | £554 | £554 | [4] |
|  |  | Speech and language x4 | £335 | £335 | [4] |
|  |  | Community team x6 | £1,012 | £1,012 | [4] |
|  |  | Telephone contact per month with community team x 12 | £84 | £84 | [4] |
|  |  | Endocrinologist | £242 | £144 | [4] |
|  |  | Neurologists x4 | £1,209 | £697 | [4] |
|  |  | **Total (annual)** | **£5,436** | **£3,855** |  |
|  | Social care costs | Education Costs | £23,966 |  | [9] |
|  |  | Care package 6 to 24 hours per week (6 hours) | £7,488 | £7,488 | [6] |
|  |  | **Total (annual)** | **£11,056** | **£7,488** |  |
| Moderate/Severe | Health care costs | Gastrostomy surgery | £2,062 | £894 | [4] |
|  |  | Gastronomy dietician x 6 appointments per year | £247 | £247 | [4] |
|  |  | Gastrostomy feeds -dependent on age | £4,000-£9,000 | | [10] |
|  |  | Hearing test | £77 |  | [4] |
|  |  | Eyesight test | £100 |  | [4] |
|  |  | Occupation therapist x two visits per year | £128 | £128 | [4] |
|  |  | 6 neurology appointment per year | £1,813 | £1,046 | [4] |
|  |  | Orthotics appointments x 2 visits per year and braces for back, splints for feet – reviews ongoing as child grows | £219 | £219 | [4] |
|  |  | Endocrinologist | £242 | £144 | [4] |
|  |  | Psychology x4 | £707 |  | [4] |
|  |  | Epilepsy management x3 visits per year | £579 |  | [4] |
|  |  | Physiotherapist x 24 visits per year | £1,108 | £1,108 | [4] |
|  |  | 4 visits per year to speech and language therapist | £335 | £335 | [4] |
|  |  | 1 face-to-face visit per month by community team | £2,024 | £2,024 | [6] |
|  |  | 1 visit per fortnight by community team | £4,047 | £4,047 | [6] |
|  |  | 1 telephone contact per fortnight with community team | £181 | £181 | [6] |
|  |  | One hospital episode for chest infection/pneumonia | £3,753 | £3,752.70 | [4] |
|  |  | **Total (annual)** | **£17,621** | **£14,125** |  |
|  | Social care costs | Education Costs | £23,966 |  | [9] |
|  |  | Uses wheelchair and has home equipment | £6,678 | £6,678 | [6] |
|  |  | 30 days of respite care | £32,098 | £32,098 | [6] |
|  |  | Care package 40 hours per week < 18's and 65 hours per week > 18's | £49,920 | £81,120 | [6] |
|  |  | **Total (annual)** | **£112,662** | **£119,896** |  |
| Cost of transplant | Average transplantation costs (paediatric) - includes two MRIs for post-transplant | Related marrow (30%) | £58,336 |  | [4] |
|  |  | Unrelated marrow (17%) | £97,454 |  | [4] |
|  |  | Cord blood (53%) | £120,138 |  | [4] |
|  |  | **Total (adjusted for proportion of different types of transplants)** | **£98,147** |  |  |

*Prices were given in euros in the paper. Converted back to GBP using the exchange rate given in the paper (1 euro to 0.6888 GBP) and then uplifted to 2014/15 prices

Reference List

1. Bessey A, Chilcott J, Pandor A, Paisley S. The cost-effectiveness of expanding the NHS Newborn Bloodspot Screening Programme to include homocystinuria (HCU), maple syrup urine disease (MSUD), glutaric aciduria type 1 (GA1), isovaleric acidaemia (IVA), and long-chain hydroxyacyl-coa dehydrogenase deficiency (LCHADD). Value In Health 2014;17**:**A531.

2. Sheffield Children's NHS Foundation Trust: Clinical Chemistry and Sheffield Diagnostic Genetic Service. User's Handbook for Metbolic Investigations. 2015.

3. NHS UK Genetic Testing Network. Find a Test. 2015. <https://ukgtn.nhs.uk/find-a-test/>. Accessed 16^th^ October 2015

4. Department of Health. NHS Reference Costs 2014 to 2015. 2015. <https://www.gov.uk/government/publications/nhs-reference-costs-2014-to-2015>. Accessed 24^th^ November 2015

5. University Hospital of Wales: Department of Medical Biochemistry and Immunology. Endocrine Laboratory Test Repertoire 2015/16. 2015.

6. Curtis L. Unit costs of health and social care 2014. Personal Social Services Research Unit, University of Kent, Canterbury; 2014

7. Kobelt G, Berg J, Lindgren P, Kerrigan J, Russel N, Nixon R. Costs and quality of life of multiple sclerosis in the United Kingdom**.** European Journal of Health Economics 2006;7**:** Suppl 5:S96-S104.

8. Kobelt G, Berg J, Lindgren P, Fredrikson S, Jönsson B. Costs and quality of life of patients with multiple sclerosis in Europe**.** J Neurol Neurosurg Psychiatry 2006;77**:**918.

9. Barrett B, Mosweu I, Jones C RG, Charman T, Baird G, Simonoff E et al. Comparing service use and costs among adolescents with autism spectum disorders, special needs and typical development**.** Autism 2015;19:5

10. Paediatric Formulary Committee. British National Formulary for Children 2014-2015. BMJ Group, Pharmaceutical Press, and RCPCH Publications; 2015
